# Functional and molecular dissection of HCMV long non-coding RNAs

**DOI:** 10.1038/s41598-022-23317-3

**Published:** 2022-11-11

**Authors:** Sungwon Lee, Hyewon Kim, Ari Hong, Jaewon Song, Sungyul Lee, Myeonghwan Kim, Sung-yeon Hwang, Dongjoon Jeong, Jeesoo Kim, Ahyeon Son, Young-suk Lee, V. Narry Kim, Jong-seo Kim, Hyeshik Chang, Kwangseog Ahn

**Affiliations:** 1 School of Biological Sciences, Seoul National University, Seoul, 08826 Republic of Korea; 2 Institute for Basic Science, Center for RNA Research, Seoul, 08826 Republic of Korea; 3 Interdisciplinary Program in Bioinformatics, Seoul National University, Seoul, 08826 Republic of Korea; 4 Department of Bio and Brain Engineering, Korea Advanced Institute of Science and Technology (KAIST), Daejeon, 34141 Republic of Korea

**Keywords:** Virology, Long non-coding RNAs, RNA modification

## Abstract

Small, compact genomes confer a selective advantage to viruses, yet human cytomegalovirus (HCMV) expresses the long non-coding RNAs (lncRNAs); RNA1.2, RNA2.7, RNA4.9, and RNA5.0. Little is known about the function of these lncRNAs in the virus life cycle. Here, we dissected the functional and molecular landscape of HCMV lncRNAs. We found that HCMV lncRNAs occupy ~ 30% and 50–60% of total and poly(A)+viral transcriptome, respectively, throughout virus life cycle. RNA1.2, RNA2.7, and RNA4.9, the three abundantly expressed lncRNAs, appear to be essential in all infection states. Among these three lncRNAs, depletion of RNA2.7 and RNA4.9 results in the greatest defect in maintaining latent reservoir and promoting lytic replication, respectively. Moreover, we delineated the global post-transcriptional nature of HCMV lncRNAs by nanopore direct RNA sequencing and interactome analysis. We revealed that the lncRNAs are modified with N^6^-methyladenosine (m^6^A) and interact with m^6^A readers in all infection states. In-depth analysis demonstrated that m^6^A machineries stabilize HCMV lncRNAs, which could account for the overwhelming abundance of viral lncRNAs. Our study lays the groundwork for understanding the viral lncRNA–mediated regulation of host-virus interaction throughout the HCMV life cycle.

## Introduction

Human cytomegalovirus (HCMV), a member of β-herpesvirus family, is an ubiquitous pathogen that infects ~ 80% of the global population^1^. HCMV establishes lifelong latency and infections are asymptomatic in most healthy people, but the virus can be reactivated and cause severe morbidity and mortality in immunocompromised patients^2^. Undifferentiated hematopoietic progenitor cells (HPCs) are the primary reservoirs for latent HCMV, showing inhibited viral gene expression and infectious particle production. During reactivation, HCMV enters a lytic infection state with cellular differentiation into macrophages or dendritic cells, wherein viral genes are highly expressed and progeny viruses are produced^3^. Viral dissemination is facilitated by infection of endothelial cells, epithelial cells, and fibroblasts, wherein lytic replication is most extensively activated^4^. Although HCMV lytic replication has been widely studied, recent research has focused on how latency and reactivation are achieved using CD34+ primary HPCs, CD14+ monocytes, or myeloid progenitor cell lines such as Kasumi-3 cells^5,6^. These experimental infection models have greatly contributed to elucidating the regulatory mechanisms that control HCMV latency and lytic replication.

HCMV virion structure consists of an outer lipid bilayer membrane, a middle proteinaceous tegument matrix, and an icosahedral nucleocapsid containing ~ 230 kb of double stranded DNA^7^. HCMV DNA encodes not only viral proteins or microRNAs (miRNAs; ~ 22 nt), but also long non-coding RNAs (lncRNAs; > 200 nt), RNA1.2, RNA2.7, RNA4.9, and RNA5.0. The number included in the name of each lncRNA indicates transcript length (kb) of the corresponding lncRNA. Although HCMV expresses ~ 200 protein coding mRNAs, these four lncRNAs are abundantly expressed during lytic replication^8^. Recent studies have revealed that latent HCMV transcripts are expressed at low levels, but the overall latent transcriptome largely resembles the lytic transcriptome, indicating that the lncRNAs are more highly expressed than other viral transcripts in latently infected cells^9,10^. Although viral lncRNAs are crucial regulatory factors involved in viral replication, persistence, and immune evasion^11^, the roles of HCMV lncRNAs throughout viral life cycle are largely unexplored. Only a few studies have reported the function of HCMV lncRNAs exclusively using lytic infection models, which do not reveal insights regarding roles in latency or reactivation^12–14^. Thus, functional investigation of HCMV lncRNAs should be comprehensively investigated in the entire viral life cycle.

The major obstacle to studying the functional role of HCMV lncRNAs is that their molecular characteristics have been barely investigated. The functions of lncRNAs are closely related to their individual molecular features, which are determined by cooperating with host post-transcriptional processing machineries^15,16^. Several lncRNAs undergo RNA modifications, such as N^6^-methyladenosine (m^6^A) RNA methylation, which determines the abundance and function of the corresponding lncRNA^17,18^. Additionally, specific characteristics of lncRNAs largely depends on their interacting proteins. To identify the functional binding proteome of lncRNAs, various ribonucleoprotein (RNP) complex pull-down strategies, including comprehensive identification of RNA binding proteins by mass spectrometry (ChIRP-MS) and RNA antisense purification coupled with mass spectrometry (RAP-MS), have been developed^19,20^. These molecular analyses have been utilized to characterize not only cellular lncRNAs but also viral lncRNAs, allowing us to understand the nature of lncRNAs during viral infection^21,22^.

In this study, we used combinational approaches to globally characterize HCMV lncRNAs. We revealed that RNA1.2, RNA2.7, and RNA4.9, but not RNA5.0, are required for lytic replication and maintenance of latent reservoirs. Moreover, we identified the comprehensive molecular features of RNA1.2, RNA2.7, and RNA4.9, including RNA modification landscape and global binding proteome. In particular, we found that m^6^A acted as a housekeeping modification of the lncRNAs in all infection states and stabilized the three lncRNAs to maintain their high expression level.

## Results

### HCMV lncRNAs occupy a significant portion of the viral transcriptome during latency, reactivation, and lytic replication

**Figure 1:**
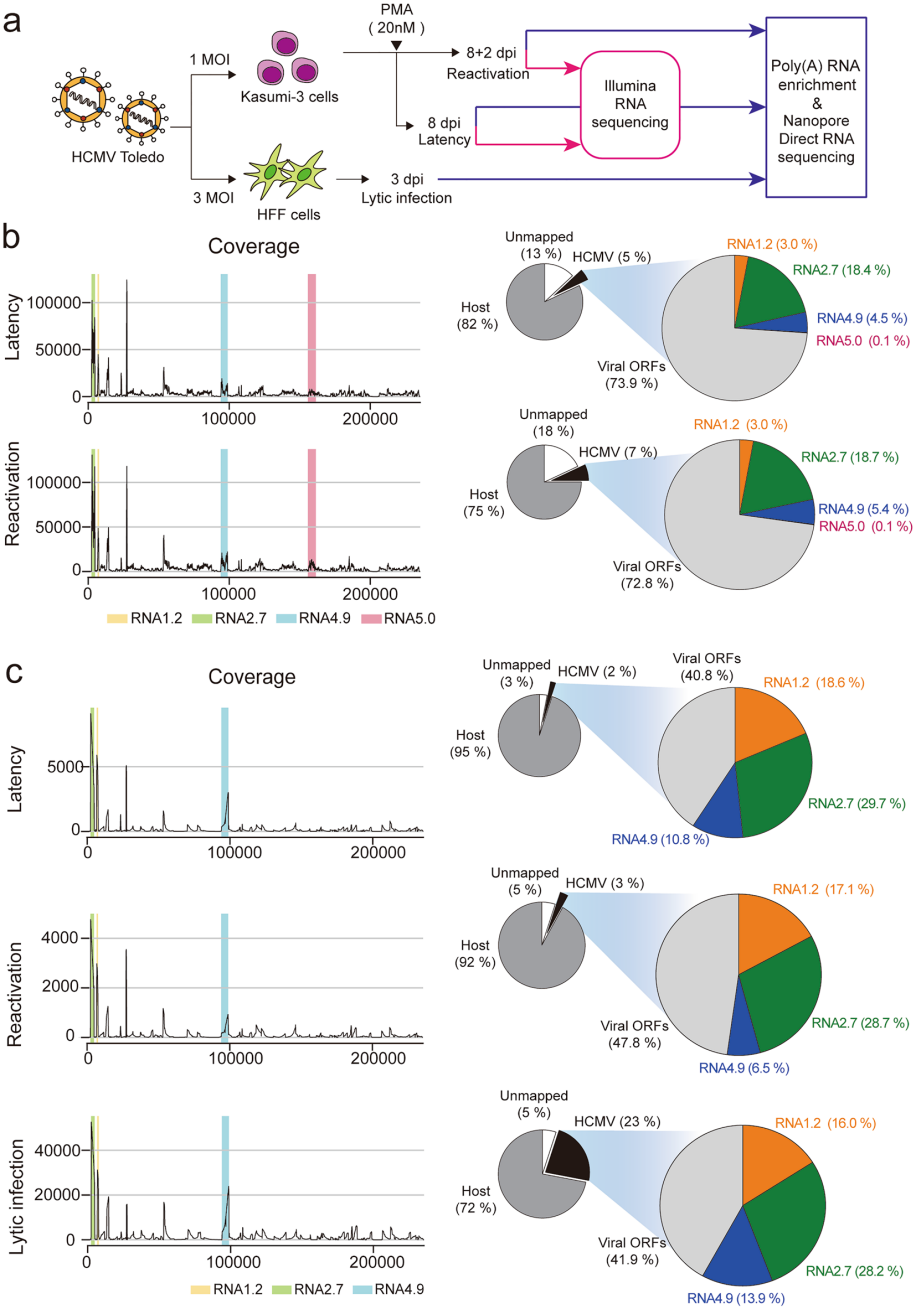
Profiling of HCMV lncRNAs by Illumina sequencing and nanopore DRS (**a**) Schematic of the sequencing strategies for profiling HCMV lncRNAs in various infections state. (**b**) Coverage of HCMV genome mapped reads (left) and statistics of read counts (right) from RNA-seq in latency and reactivation. (**c**) Coverage of HCMV genome mapped reads (left) and statistics of read counts (right) from nanopore DRS in latency, reactivation, and lytic infection stages.

To quantitatively analyze the expression of HCMV lncRNAs across the viral life cycle, we performed RNA sequencing (RNA-seq) using latent and reactivated viral samples (Fig. 1a). Kasumi-3 cells were infected with HCMV Toledo at a multiplicity of infection (MOI) of 1, and harvested at 8 days post infection (dpi) as a latency sample. Then the cells were treated with phorbol 12-myristate 13-acetate (PMA) for 2 days and harvested as a reactivation sample. To confirm that the sequencing samples reflected each infection state, we compared our RNA-seq data from the latency sample with previously reported data, forming a high-resolution latent transcriptome in CD34+ HPCs through viral gene enrichment using targeted probes (SureSelect; SS, Agilent)^23^. We observed that the expression of protein-coding viral genes in our latency sample was highly correlated with the previous data obtained from both experimental and clinical latency samples, although these excluded HCMV lncRNAs from target enrichment (Supplementary Fig. 1a). Furthermore, RNA-seq data showed that most viral genes, including major immediate early genes UL122 (IE2) and UL123 (IE1), were upregulated in reactivation than latency (Supplementary Fig. 1b, Supplementary Table 1). These results indicate that our latency and reactivation samples well described each infection state. In the RNA-seq reads, 5% and 7% of total reads were mapped to the HCMV genome during latency and reactivation, respectively (Fig. 1b). Of note, we found that four HCMV lncRNAs occupied a considerable proportion (~ 30%) of the total viral transcriptome during both states (Fig. 1b). Among the viral transcripts, RNA2.7 occupied the largest portion (~ 18%) of the transcriptome, whereas RNA1.2 and RNA4.9 accounted for approximately 3% and 5%, respectively. The expression of RNA5.0 was much lower (0.1%) than that of other HCMV lncRNAs (Fig. 1b).

Next, we used nanopore direct RNA sequencing (DRS), which is selective for poly(A)+ transcripts, to compare the expression of HCMV lncRNAs to that of viral mRNAs (Fig. 1a). In addition to the latency and reactivation samples, we included lytic infection sample for nanopore DRS. For lytic infection sample, human foreskin fibroblasts (HFFs) were infected with HCMV Toledo at a MOI of 3 and harvested at 3 dpi (Fig. 1a). The results showed that most viral genes were upregulated in the reactivation and lytic infection samples compared to expression in the latency sample (Supplementary Fig. 1c, Supplementary Table 1). Among total DRS reads, 2–3% from latency and reactivation samples and approximately 25% from lytic infection samples were mapped to the HCMV genome, reflecting robust viral gene expression during lytic replication in HFFs compared to those during other infection states (Fig. 1c). DRS results revealed that RNA1.2, RNA2.7, and RNA4.9 account for 50–60% of poly(A)+ viral transcriptome at all infection states, although RNA5.0 was not included due to its lack of poly(A) tail^24^ (Fig. 1c). Among the poly(A) viral transcripts, RNA2.7 was the most abundant, accounting for 29% of the total transcriptome; RNA1.2 and RNA4.9 accounted for approximately 17% and 6–13%, respectively. Therefore, we observed that HCMV lncRNAs occupied more than half of the poly(A)+ viral transcriptome in all infection states, suggesting potential roles of HCMV lncRNAs throughout the overall viral life cycle.

### RNA1.2, RNA2.7 and RNA4.9, but not RNA5.0, are necessary for productive lytic replication

**Figure 2:**
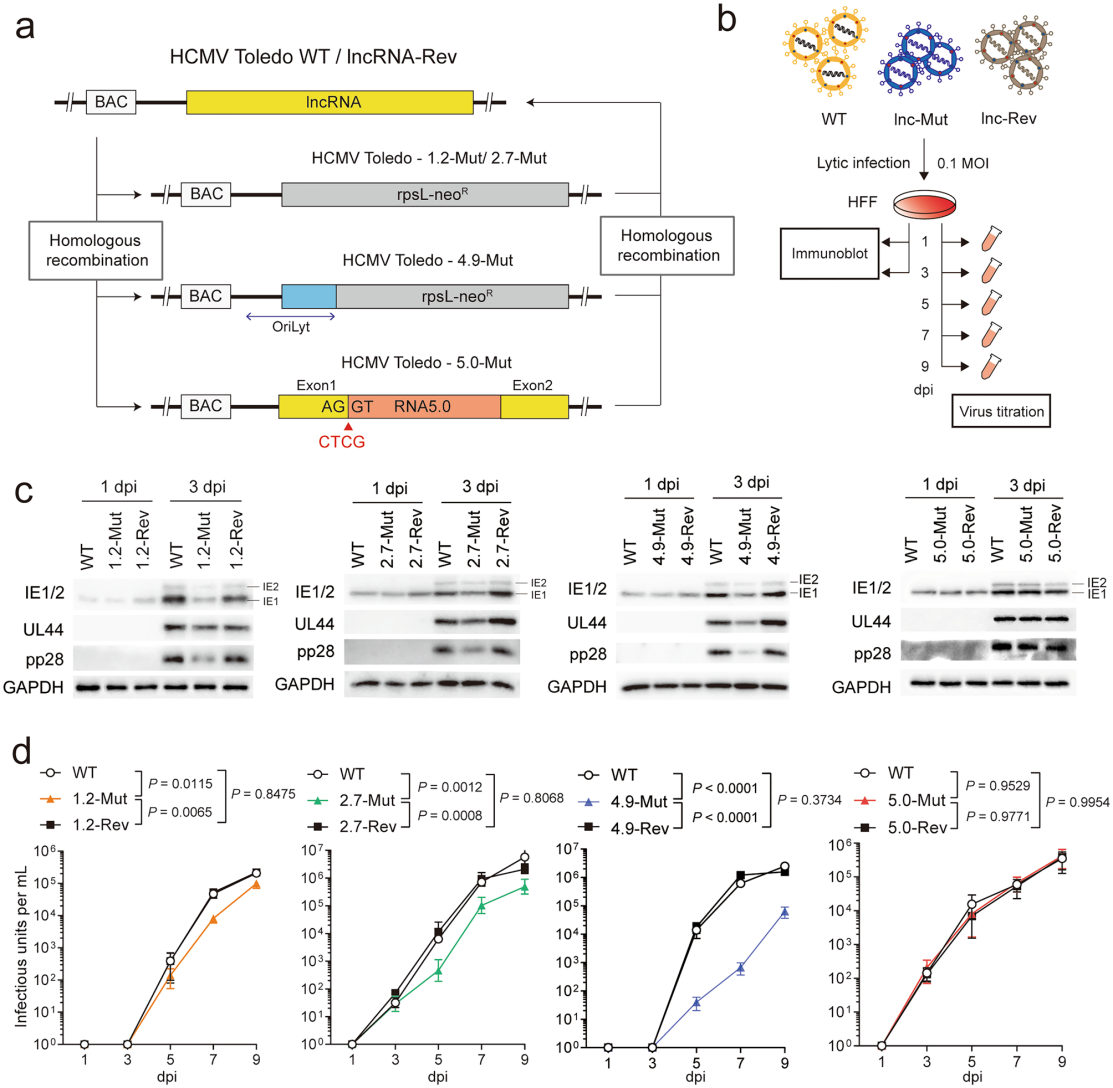
HCMV lncRNAs other than RNA5.0 are required for lytic replication in HFFs. (**a**) Schematic representation for generation of HCMV lncRNA-Mut virus. (**b**) Schematic representation of lytic infection analysis in HFFs using WT, lncRNA-Mut, and lncRNA-Rev viruses. (**c**) Immunoblot assay of viral proteins, which indicate the lytic replication phase (IE1/2, Immediate early; UL44, early; pp28, late). GAPDH served as a loading control. Images are representative of two independent experiments. (**d**) Cell-free viral particles from supernatant medium were titrated via limiting dilution assay. Data represents mean ± SEM, and the statistical significance was calculated by two-way ANOVA with Tukey’s multiple comparisons test (n = 3).

To directly investigate the function of HCMV lncRNAs, we modified the HCMV Toledo bacterial artificial chromosome (BAC) strain (GenBank AC146905.1) by recombineering and generated a mutant virus without expression of each lncRNA (hereafter referred to as lncRNA-Mut) (Fig. 2a). For RNA1.2 and RNA2.7, the genomic region of each lncRNA was completely deleted. For the 4.9-Mut virus, we could not delete the whole genomic region of RNA4.9, as the 5′ sequence of RNA4.9 overlaps with lytic origin of replication (*oriLyt*)^25^. Instead, we deleted the ~ 3 kb region from the 3′ end of the RNA4.9 sequence, leaving the essential and accessory region of *oriLyt*^26,27^. Because RNA5.0 is a stable intron, we modified the splice donor site and prevented subsequent splicing from pre-mRNA to generate 5.0-Mut HCMV, as in a previous report^24^. Each mutation in the lncRNA region was reversed by introducing the original sequence to generate a revertant virus (hereafter referred to as lncRNA-Rev). After recombination, we transfected each recombinant BAC into HFFs and propagated viruses.

We then determined whether full-length lncRNA was expressed in the lncRNA-Mut virus. Northern blot analysis revealed that the expression of each full-length lncRNA was completely depleted in the lncRNA-Mut virus, but not in the WT and lncRNA-Rev viruses (Supplementary Fig. 2a). Furthermore, the depletion of lncRNA expression in each mutant virus was confirmed by reverse transcription-quantitative PCR (RT-qPCR) and single-molecule fluorescence in situ hybridization (smFISH) (Supplementary Fig. 2b and c). In case of smFISH, immediate early proteins (IE1/2) that served as infection controls were subsequently stained by immunofluorescence assay (IFA). In the WT virus, only RNA4.9 formed large speckles representing the viral replication complex (VRC) in the nucleus, whereas RNA1.2, RNA2.7, and RNA5.0 were predominantly localized to the cytoplasm, as described previously^12^. We did not detect any lncRNA signals in the lncRNA-Mut virus-infected cells, indicating depletion of lncRNA expression in this virus (Supplementary Fig. 2c).

Next, we examined the effects of HCMV lncRNA defects on productive lytic replication. We found no significant difference between viruses in terms of entry into HFFs (Supplementary Fig. 2d). Then the cells were harvested at the indicated time points to examine viral protein levels (Fig. 2b). Notably, immunoblot analysis showed that protein levels of immediate early (IE1/2), early (UL44), and late (pp28) genes were substantially reduced in the 1.2-Mut, 2.7-Mut, and 4.9-Mut viruses compared to levels in WT, especially at 3 d post-infection (Fig. 2c). However, the 5.0-Mut virus showed viral protein levels comparable to those of WT (Fig. 2c). Each lncRNA-Rev (1.2-Rev, 2.7-Rev, 4.9-Rev) fully complemented the lncRNA-Mut virus phenotype, confirming that these results did not occur due to aberrations in the viral genome.

We then measured the titer of cell-free virus over the course of infection (Fig. 2b). In consistent with viral protein levels, 1.2-Mut, 2.7-Mut, and 4.9-Mut produced fewer progeny viruses than WT during lytic replication, but 5.0-Mut did not (Fig. 2d). Either 1.2-Mut or 2.7-Mut showed a slight reduction (2–10 fold) in virus production compared to that of WT. In particular, 4.9-Mut showed the greatest reduction in progeny virus production (20–100 fold), indicating differential role of each HCMV lncRNA during lytic replication (Fig. 2d). Each lncRNA-Rev (1.2-Rev, 2.7-Rev, 4.9-Rev) showed similar virus production level compared to WT. Therefore, we conclude that HCMV lncRNAs, except RNA5.0, actively contribute to viral gene expression and virus production during lytic replication.

### RNA1.2, RNA2.7 and RNA4.9, but not RNA5.0, are required for maintenance of the latent reservoir and for subsequent reactivation

We then investigated function of HCMV lncRNAs during viral latency. To establish an experimental latency model, we infected Kasumi-3 cells with HCMV Toledo at an MOI of 1, harvested the cells throughout the time course of infection, and measured the viral DNA and RNA levels. During HCMV latency, viral DNA is maintained, but lytic viral gene expression is repressed^3,5^. The viral DNA level of the major immediate early gene (UL123) increased, but its RNA level decreased over the course of the infection, indicating successful latency establishment (Supplementary Fig. 3a). Furthermore, we confirmed that infected Kasumi-3 cells produced more viral particles upon reactivation with PMA, implying that the experimental model reflected cell differentiation-mediated viral reactivation (Supplementary Fig. 3b). These results were consistent with those of previous reports showing that the HCMV Toledo strain, like other low-passage clinical strains, can establish latent infections in myeloid progenitor cells^6,28^.

**Figure 3:**
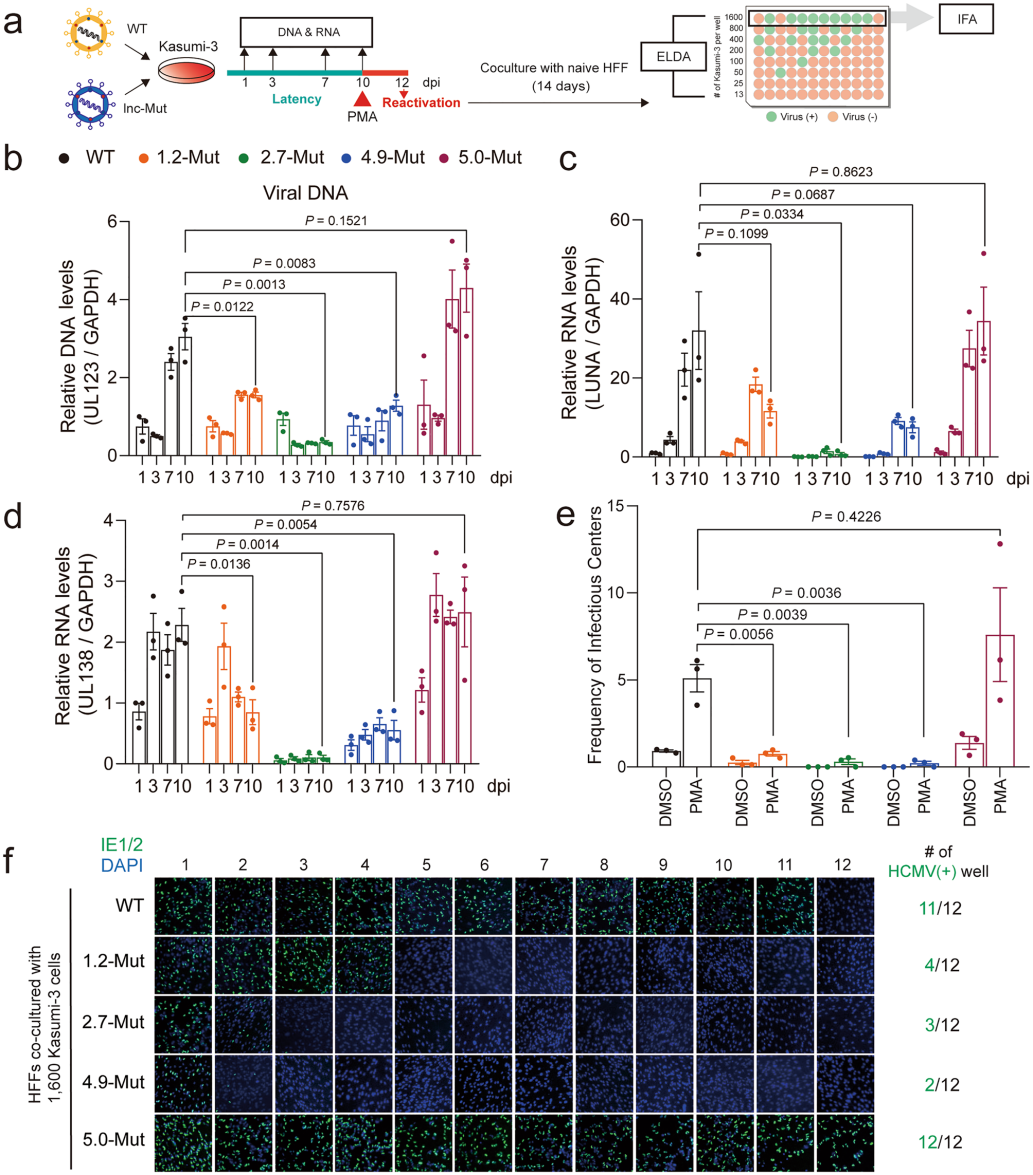
HCMV lncRNAs other than RNA5.0 are necessary for latent reservoir maintenance and efficient reactivation in Kasumi-3 cells. (**a**) Schematic representation of latency and reactivation analysis in Kasumi-3 cells using WT and lncRNA-Mut viruses. (**b**) The levels of viral DNA at the indicated time points were measured by qPCR of the UL123 region, and normalized against GAPDH. (**c**, **d**) The RNA levels of LUNA (**c**), and UL138 (**d**) at the indicated time points were quantified using qRT-PCR. (**e**) WT or lncRNA-Mut HCMV-infected Kasumi-3 cells were treated with DMSO or 20 nM PMA and co-cultured with naïve HFF cells. Frequency of infectious centers was calculated using ELDA. (**f**) Representative images of HFF cells co-cultured with 1600 PMA-treated Kasumi-3 cells for 14 days. Each image is obtained from each well of HFFs. IE1/2-positive images are presented on the left. Merged images are shown for IE1/2 (green), and DAPI (blue) staining. (**b**–**e**) Data represent mean ± SEM. All experiments were performed independently (n = 3), and the statistical significance was calculated by two-tailed unpaired *t*-test.

To determine whether HCMV lncRNAs are necessary for maintaining viral latency, we infected Kasumi-3 cells with WT and lncRNA-Mut HCMV Toledo at an MOI of 1, after which viral DNA and RNA levels were analyzed during the course of infection (Fig. 3a). We found that the degree of entry of each virus into Kasumi-3 cells was similar (Supplementary Fig. 3c). Notably, the 1.2-Mut, 2.7-Mut, and 4.9-Mut viruses did not efficiently maintain viral DNA during latent infection, whereas the 5.0-Mut virus showed similar viral DNA levels to those of the WT (Fig. 3b). One interesting point is that, unlike lytic infection, the 2.7-Mut virus showed the most dramatic decrease in viral DNA levels than WT virus during latency. These results suggest potential different roles of HCMV lncRNAs between in latency and lytic infection. The 1.2-Rev, 2.7-Rev, and 4.9-Rev viruses showed comparable viral DNA levels to those of the WT virus in latency, indicating that the latent phenotype of each mutant virus did not arise due to a defect in the viral genome (Supplementary Fig. 3d).

We next measured the RNA levels in each virus during latency. Several viral transcripts, such as LUNA and UL138, exhibit higher expression levels than other lytic genes in latency, which is required for the establishment of HCMV latency or further reactivation^29,30^. Levels of LUNA and UL138 RNA were highly maintained in the WT virus during latency (Fig. 3c and d), in contrast to the decreased RNA level of UL123 (Supplementary Fig. 3a). Of note, RNA levels of LUNA and UL138 were significantly lower in 1.2-Mut, 2.7-Mut, and 4.9-Mut viruses than in the WT virus, but were comparable in 5.0-Mut virus (Fig. 3c and d). RNA levels of LUNA and UL138 were most dramatically reduced in 2.7-Mut virus infection, indicating that decreased viral DNA levels of the lncRNA-Mut virus resulted in reduced viral RNA levels.

We next explored whether the decreased latent reservoir of the lncRNA-Mut virus impaired viral reactivation. To compare infectious viral particle production during reactivation, WT and lncRNA-Mut HCMV Toledo-infected Kasumi-3 cells were treated with DMSO or PMA 10 days after infection. After 2 d, the cells were co-cultured with HFFs for 14 d. The abundance of infectious particles was analyzed using an extreme limiting dilution assay (ELDA) (Fig. 3a). Production of 1.2-Mut, 2.7-Mut, and 4.9-Mut viruses, which do not efficiently maintain latent reservoirs, was dramatically reduced compared to that of WT during reactivation, but that of 5.0-Mut was not (Fig. 3e). We then performed IFA using IE1/2 protein antibodies in HFFs co-cultured with infected Kasumi-3 cells (Fig. 3a). This assay showed that the frequency of infectious centers in HFFs co-cultured with 1.2-Mut, 2.7-Mut, and 4.9-Mut, but not 5.0-Mut virus-infected Kasumi-3 cells was substantially lower than that of HFFs co-cultured with the WT virus infected Kasumi-3 cells (Fig. 3f). In addition to progeny virus production, 1.2-Mut, 2.7-Mut, and 4.9-Mut exhibited reduced UL123 RNA levels during reactivation (Supplementary Fig. 3e). Altogether, these results suggested that HCMV lncRNAs other than RNA5.0 were critical for the maintenance of latent reservoirs and subsequent viral reactivation.

### The epitranscriptome landscape of RNA1.2, RNA2.7 and RNA4.9 throughout HCMV life cycle

Unlike conventional short-read RNA-seq, nanopore DRS directly reads the native RNA molecules instead of cDNA, enabling the detection of epitranscriptomic features, such as the poly(A) tail status and RNA base modifications of full-length transcript isoforms^31–34^. To understand the detailed molecular features of RNA1.2, RNA2.7, and RNA4.9, we explored whether viral lncRNAs expressed multiple alternatively spliced transcript isoforms. Although the data confirmed the known canonical splicing event in the UL36 reads (Supplementary Fig. 4a, gray), no splicing junction was detected within RNA1.2, RNA2.7, or RNA4.9, indicating that these lncRNAs were expressed only in unspliced full-length isoforms (Supplementary Fig. 4a). We then measured the poly(A) tail length of the viral RNAs directly from the low-variance ionic current signals using Nanopolish^35^. Throughout all infection states, the median poly(A) length of RNA1.2, RNA4.9, and other 22 viral mRNAs varied from 87 to 90, 90–93, and 100–110 nt, respectively (Supplementary Fig. 4b). DRS results showed that RNA2.7 exhibited a much longer poly(A) tail length (120–150 nt) than other viral transcripts throughout viral life cycle, which accounts for the highest expression level of RNA2.7 among viral transcriptome (Fig. 1b and c).

RNA base modification is a critical post-transcriptional event that controls the fate of both viral and cellular RNA. Dynamic RNA modifications of viral transcripts are required to regulate the viral life cycle^36,37^. To analyze the differential RNA modification landscape of the HCMV transcriptome throughout the viral life cycle, we used ELIGOS2, an RNA modification analysis tool based on differential error rates^38^. ELIGOS2 compares the error rates in the control and test samples at each position to yield an odds ratio of the enriched errors. Thus, a higher odds ratio may reflect a higher chance of RNA modification at this position. Comparisons among latency, reactivation, and lytic infection samples were performed for pairs of any two samples.

**Figure 4:**
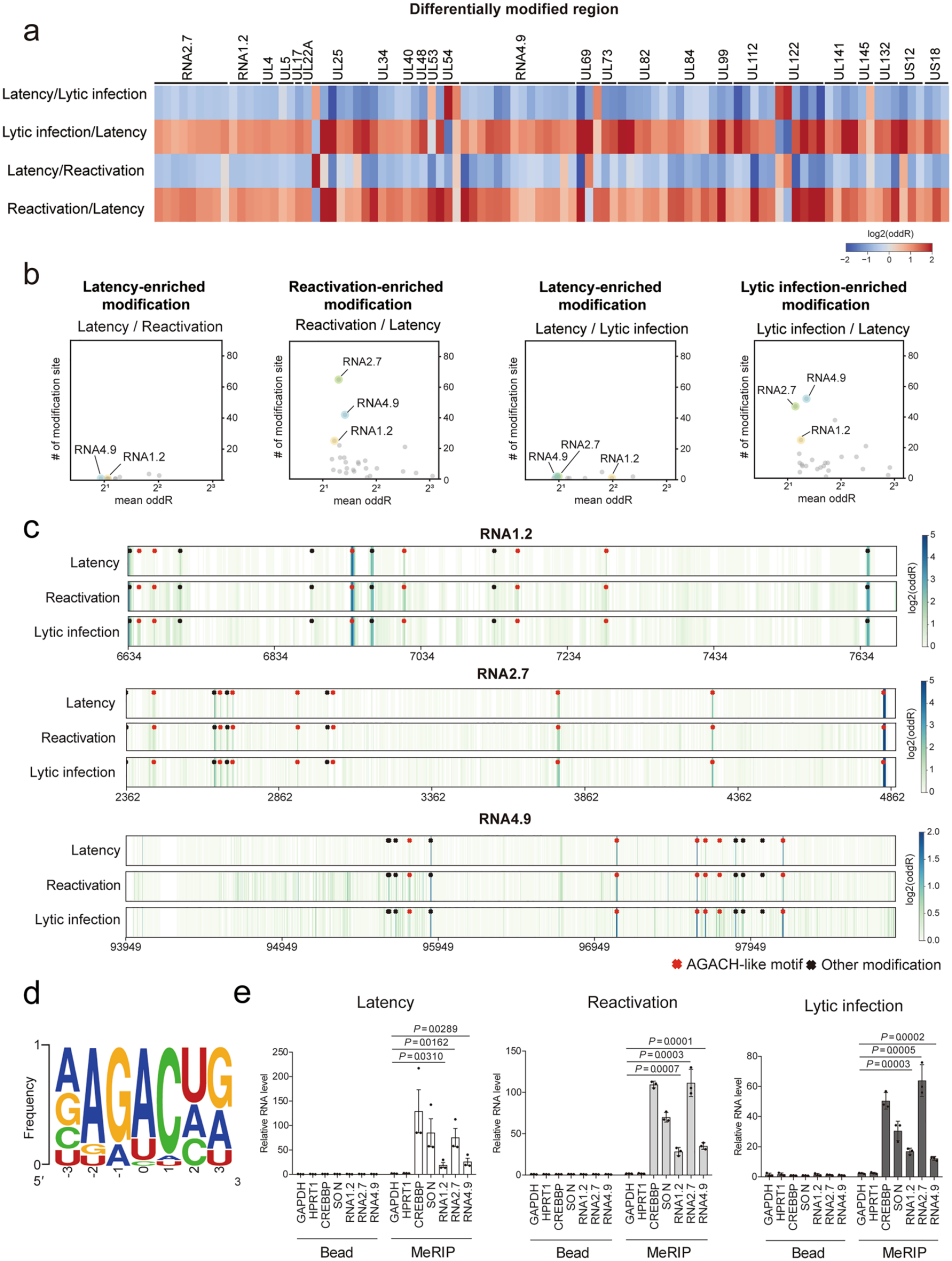
Post-transcriptional landscape of HCMV transcripts throughout the viral life cycle. (**a**, **b**) Differentially modified regions in viral transcripts. In comparison of test and control groups (test/control), enriched modification score (odds ratio) was analyzed by ELIGOS2. (**a**) Viral transcripts were divided into 300 nt tiles, and the median log_2_ odds ratio for each tile is presented as a heatmap. (**b**) Scatter plot showing the relationship between number of differential modification sites (odds ratio > 1.8) and average odds ratio for each gene. (**c**) Location and modification levels of RNA1.2, RNA2.7, and RNA4.9 in latency, reactivation, and lytic infection. Putative modification sites (odds ratio > 1.8) with AGACH motifs (red) and the others (black) are marked. (**d**) Position-specific base frequency of a motif enriched in the putative modified sites. (**e**) MeRIP-qPCR of latency, reactivation, and lytic infection samples. HPRT1 and GAPDH served as m^6^A-negative controls. CREBBP and SON served as m^6^A-positive controls. Data represent mean ± SEM. All experiments were performed independently (n = 3), and the statistical significance was calculated by two-tailed unpaired *t*-test.

Overall, we identified differentially modified regions in viral RNAs during the viral life cycle (Fig. 4a, Supplementary Table 2). Interestingly, we found that most regions in the viral transcripts showed a lower error rate in latency than in either reactivation or lytic infection (Fig. 4a). The number of latency-enriched modified sites was lower than that of reactivation- or lytic infection–enriched sites, indicating decreased RNA modifications of the viral transcripts only in latency (Fig. 4b). Of note, differentially modified sites between latency and one of the other infection states were far more frequent in viral lncRNAs (RNA1.2, RNA2.7, and RNA4.9) than in viral mRNAs. (Fig. 4b). These results suggested that these three lncRNAs were the predominant targets of post-transcriptional regulation by RNA modification across the viral life cycle.

### RNA1.2, RNA2.7, and RNA4.9 are modified with m^6^A in all states of infection

To unambiguously define the modification sites of RNA1.2, RNA2.7, and RNA4.9, we sequenced HCMV lncRNAs transcribed in vitro as unmodified controls using nanopore DRS. The in vitro transcripts (IVT) control library comprised transcripts covering the entire length of each lncRNA and partially overlapping, each measuring 1–2 kb in length (Supplementary Fig. 5a). To equalize the sensitivity of detection over all positions, the sequenced reads in each sample were subsampled to yield balanced coverage for each lncRNA (Supplementary Fig. 5b). We obtained sufficient depth covering all positions in the three lncRNAs, except for roughly 1 kb of the 5 region of RNA4.9, which was highly GC-rich. Using the IVT control, we screened the high-confidence modification sites by comparing differential error rates with ELIGOS2 software. We found that 36 sites showed a substantially higher level of errors in viral lncRNAs than in unmodified IVT controls, which represents potential RNA modification (Fig. 4c). The posterior probabilities for base calling decreased near the putative modification sites only in viral infection samples and not in the IVT sample, suggesting that RNA modifications distorting the ionic current signal were unique to the HCMV-derived RNAs (Supplementary Fig. 6). Fifteen of the 36 sites exhibited inconsistent levels of error enrichment among the viral infectious states (Supplementary Fig. 7). These sites were the least modified in the latency sample across the viral life cycle (6649, 6670, 6885, 7134, and 7166 for RNA1.2; 3018 and 3037 for RNA2.7; 95627, 95633, 95675, 95764, 97656, 97747, 97897, and 98021 for RNA4.9). In summary, by comparing native lncRNA signals to those of IVT controls, we found 15 sites that were differentially regulated according to infection states and 21 steady modification sites shared across all infection states.

Next, we investigated whether any sequence motifs were enriched in the detected modification sites. The most significant motif within HCMV lncRNAs is ‘AGACH’ (H = A, C, or U), which is a well-known motif for eukaryotic m^6^A modification (Fig. 4d)^39–41^. Among the 36 sites, 20 contained the AGACH-like motif; six sites on RNA1.2, eight on RNA2.7, and six on RNA4.9, implying potential m^6^A modifications on all three HCMV lncRNAs (Fig. 4c and Supplementary Fig. 7). Notably, 13 of the 20 AGACH-like sites were steadily modified in all infection states (6940, 7011, and 7287 in RNA1.2; 2452, 2668, 2709, 2921, 3771, 4276, and 4834 in RNA2.7; 97089, 97603, and 98152 in RNA4.9; Supplementary Fig. 7). To validate the steady m^6^A modifications in the viral lncRNAs, we performed methyl-RNA immunoprecipitation (MeRIP) using each of the infected samples. MeRIP-qPCR data showed that the m^6^A-modified positive controls CREBBP and SON were more enriched than the m^6^A-negative controls HPRT1 and GAPDH (Fig. 4e). As detected via DRS, we observed substantially higher m^6^A enrichment for the three HCMV lncRNAs than for the m^6^A-negative controls in all infection states (Fig. 4e). Therefore, with orthogonal evidence from DRS-based detection with the IVT control and from MeRIP, we concluded that m^6^A serves as a housekeeping base modification on RNA1.2, RNA2.7 and RNA4.9 in all infection states.

### Identification of the global HCMV lncRNA interactome

Like RNA modification, the interaction between lncRNAs and RNA binding proteins (RBPs) is a critical post-transcriptional event that shapes the function of lncRNAs. To identify the global binding proteome of RNA1.2, RNA2.7, and RNA4.9, we utilized a modified RAP-MS protocol using densely overlapping 90 nt probes to capture RNA-binding proteins more robustly and sensitively^42^ (Supplementary Fig. 8a). Because the amount of RNP complex is a crucial factor in the quality of RAP-MS results, we used a lytic infection sample other than latency or reactivation samples. In addition to the sense strand of lncRNA-targeting probes, antisense strand of lncRNA-targeting probes were designed to exclude off-target RNA-binding proteins (Supplementary Table 3). No probe sample was used as a negative control to exclude the bead-binding proteins. Bead-binding RNA extraction before benzonase treatment revealed that three lncRNAs were pulled down by each sense-lncRNA targeting probe, but not by the negative control probe or by antisense lncRNA targeting probes (Supplementary Fig. 8b). Furthermore, we observed that benzonase-eluted binding proteins were enriched by sense-lncRNA targeting probes relative to levels for no probe or antisense lncRNA targeting probes (Supplementary Fig. 8c). These results indicated that modified RAP-MS efficiently captured the RNP complexes of the three HCMV lncRNAs.

All enriched binding proteins were further analyzed using liquid chromatography with tandem mass spectrometry (LC–MS/MS). To identify significant binding proteins, we first excluded bead-binding proteins that were detected in a no-probe sample (False discovery rate; FDR < 0.01). The no-probe-filtered proteins showed a high correlation between two independent replicates, indicating sufficient reproducibility of the RAP-MS procedure (Supplementary Fig. 9a). These proteins were subsequently filtered by comparison with the antisense-lncRNA-targeting sample (FDR < 0.05).

For viral proteins, multiple tegument proteins are identified as significant binding proteins for HCMV lncRNAs including IRS1, UL35, and UL47, which are involved in immune evasion or virion assembly process (Supplementary Fig. 9a, Supplementary Table 4)^43–45^. Interestingly, RNA4.9, which is localized in the nucleus, bind to specific nuclear proteins, such as UL29, UL31, and UL95 (Supplementary Fig. 9a, Supplementary Table 4). These viral proteins regulate transcription of immediate-early viral genes, viral DNA-mediated antiviral responses, and formation of viral replication complex^46–48^.

**Figure 5:**
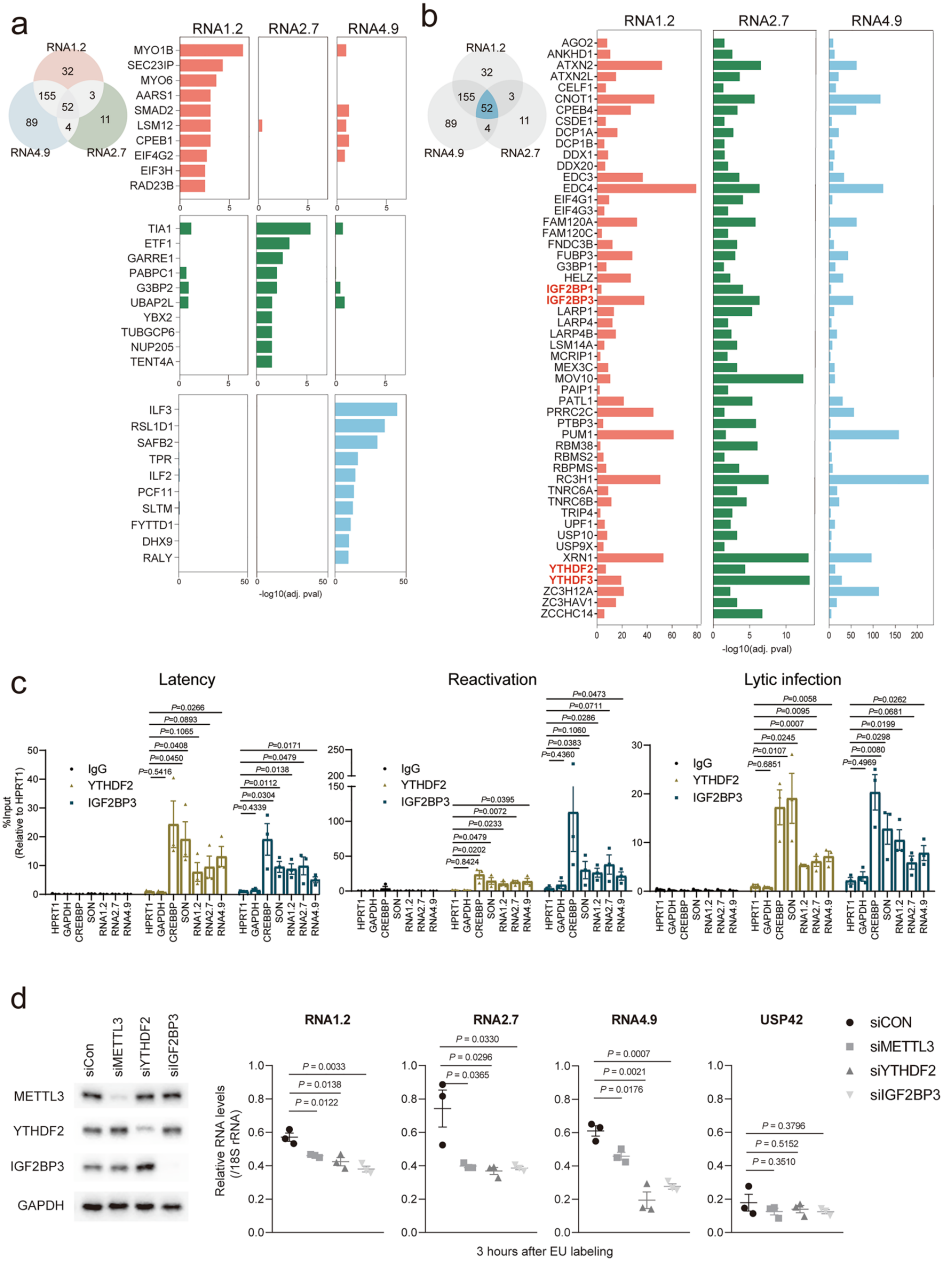
m^6^A readers interact with and stabilize RNA1.2, RNA2.7, and RNA4.9 (**a**, **b**) Significant binding proteins filtered by comparing with no probe and antisense-lncRNA targeting probe samples. Venn diagrams represent the number of significantly binding proteins. Top 10 lists of lncRNA-specific binding proteins in order of adjusted p-value are presented in (**a**), and common binding proteins of the three lncRNAs are presented in (**b**). (**c**) Enrichment of lncRNA in immunoprecipitation of YTHDF2 or IGF2BP3. HPRT1 and GAPDH served as negative controls for m^6^A modification; CREBBP and SON served as positive controls. (**d**) RNA1.2, RNA2.7, and RNA4.9 are stabilized by m^6^A readers. (Left) Protein expression of METTL3, YTHDF2, and IGF2BP3 in each gene targeting siRNA-treated HFFs. GAPDH served as a loading control. (Right) Stability of EU-labeled HCMV lncRNAs. USP42 served as a negative control. Data represents values relative to a 0 h sample. (**c**, **d**) Data represent mean ± SEM of independent experiments (n = 3); statistical significance was calculated by two-tailed unpaired *t*-test.

For human proteins, we identified 242, 70, and 300 proteins as significant binding proteins for RNA1.2, RNA2.7, and RNA4.9, respectively (Fig. 5a and b, Supplementary Table 4). To obtain comprehensive features of the lncRNA interactome, we performed gene ontology (GO) analysis of terms for non-redundant biological functions of the binding proteins. GO analysis revealed that proteins involved in posttranscriptional regulation of gene expression and RNA catabolic processes were generally captured, indicating that RAP-MS efficiently captured RNA-binding proteins (Supplementary Fig. 9b). GO analysis of the interactome in terms of cellular components revealed that proteins interacting with all three lncRNAs were involved in RNP granule formation (Supplementary Fig. 9c). Furthermore, we confirmed that a considerable portion of RNA4.9 binding proteins were localized in the nucleus, whereas RNA1.2 and RNA2.7 binding proteins were predominantly localized in the cytoplasm (Supplementary Fig. 9c), consistent with the localization signal of each lncRNA (Supplementary Fig. 2c).

To further analyze the human interactome in detail, we first selected the unique binding proteins of the three lncRNAs. Proteins that interacted with only one lncRNA at a statistically significant level (FDR < 0.05) were defined as unique binding proteins for the corresponding lncRNA. We identified 32, 11, and 89 unique proteins that specifically bound to RNA1.2, RNA2.7, and RNA4.9, respectively (Fig. 5a, Supplementary Table 4). The top 10 unique binding protein subsets per lncRNA, in order of significance, are presented in Fig. 5a. The unique binding proteins of RNA1.2 included regulators of the ER-Golgi network, such as MYO1B, MYO6, and SEC23IP, which are involved in the secretory pathway^49–51^. Among RNA2.7 unique binding proteins, we observed that TENT4A, previously identified as an RNA2.7 stabilizing protein^52^, specifically interacted with RNA2.7, but not with RNA1.2 or RNA4.9. RNA2.7 unique binding proteins also included RNA turnover machineries, such as TIA1, GARRE1, PABPC1, and G3BP2^53–56^, indicating that RNA2.7 homeostasis was specifically controlled. Moreover, unique binding proteins of RNA4.9 contained nuclear proteins, including ILF2, ILF3, RSL1D1, TPR, PCF11, SLTM, FYTTD1, and RALY^57–64^, which are involved in epigenetic regulation, RNA transcription, transcription termination, and RNA export.

Of the total interactome, 52 common binding proteins interacted with all three lncRNAs (Fig. 5b, Supplementary Table 4). Of these, 11 were involved in antiviral responses. For instance, DCP1A, DCP1B, MOV10, UPF1, XRN1, and ZC3HAV1, which exert antiviral activity by directly binding with and destabilizing viral RNAs^65–67^, were identified as common binding proteins. Furthermore, DDX1, G3BP1, HELZ, LSM14A, and MEX3C, which exhibit antiviral activity by accelerating viral RNA–mediated innate immune responses^68–72^, were also identified as common binding proteins. The commonality of these antiviral protein subsets suggested that viral lncRNAs may modulate host defense mechanisms against HCMV infection.

### m^6^A readers promote stability of RNA1.2, RNA2.7, and RNA4.9

Of the 52 common binding proteins, we found that YTHDF and IGF2BP, well-known m^6^A readers^73,74^, significantly interacted with RNA1.2, RNA2.7, and RNA4.9 (Fig. 5b). Because three lncRNAs were modified with m^6^A in all infection states (Fig. 4e), we assessed whether these m^6^A readers could bind to the three lncRNAs during latency or reactivation in Kasumi-3 cells, or during lytic infection in HFFs. To confirm this, non-cross-linked cells from latency, reactivation, and lytic infection states were subjected to RNA-immunoprecipitation (RNA-IP) using YTHDF2 and IGF2BP3 antibodies. Of note, we observed that the three lncRNAs were significantly enriched by both YTHDF2 and IGF2BP3 antibodies in all three infection states (Fig. 5c). HPRT1 and GAPDH served as negative controls, whereas CREBBP and SON served as positive controls. These results indicated that the three lncRNAs interact with m^6^A readers in all the infection states.

Because one of the major roles of m^6^A is to control RNA stability^73,74^, we examined the stability of RNA1.2, RNA2.7, and RNA4.9 in m^6^A machinery–depleted cells. HFFs were depleted with lncRNA-binding m^6^A readers YTHDF2 and IGF2BP3, and m^6^A writer METTL3^75^ and infected with HCMV at a MOI of 1. At 72 h post-infection, the cells were treated with 5′-ethynyluridine (EU) for 2 h to label nascent RNAs. Three hours after labeling, the remaining labeled RNAs were captured and the abundance of lncRNAs was measured by qRT-PCR. We confirmed that the protein expression of METTL3, YTHDF2, and IGF2BP3 was substantially reduced by each gene-targeting siRNA than by siCon (Fig. 5d; left). In control samples, we observed that the remaining labeled transcript level of RNA2.7 was higher than that of RNA1.2 and RNA4.9, implying high stability of RNA2.7 (Fig. 5d; right). This result was supported by the long poly(A) length of RNA2.7 (Supplementary Fig. 4b). Notably, remaining levels of nascent RNA1.2, RNA2.7, and RNA4.9 transcripts after EU labeling were significantly reduced in METTL3-, YTHDF2-, and IGF2BP3-depleted cells than those in control cells (Fig. 5d; right). The level of USP42, the negative control, was not changed by the knockdown of these m^6^A machineries.

Furthermore, we analyzed the stability of HCMV lncRNAs in m^6^A machineries depleted HFFs by treatment of transcription inhibitor actinomycin D. At 72 hpi, the cells were treated with the actinomycin D and harvested at the indicated time points. In the control samples, we observed that the abundance of RNA1.2 and RNA4.9 rapidly decreased after 3 h of actinomycin D treatment (Supplementary Fig. 10). However, the level of RNA2.7 remained almost consistent even after 12 h of actinomycin D treatment, indicating the high stability of RNA2.7 (Supplementary Fig. 10). We also observed that the levels of the three HCMV lncRNAs were significantly reduced in METTL3-, YTHDF2-, and IGF2BP3-depleted cells than those in control cells during actinomycin D treatment (Supplementary Fig. 10). Therefore, these data demonstrated that YTHDF2 and IGF2BP3, identified as lncRNA-binding m^6^A readers by modified RAP-MS, promote the stabilities of RNA1.2, RNA2.7, and RNA4.9.

## Discussion

In this study, we extensively characterized HCMV lncRNAs. We found that the abundantly expressed RNA1.2, RNA2.7, and RNA4.9 are necessary for lytic replication in fibroblasts and for sustaining latently-infected myeloid lineage cells harboring the potential for viral reactivation. Furthermore, by using nanopore DRS and modified RAP-MS analyses, we elucidated the dynamic molecular interplay between the three lncRNAs and host RNA-processing machineries, highlighting that the conserved m^6^A modification of the three lncRNAs in all infection states is required for stabilization of them.

Although a few roles of HCMV lncRNAs have been reported in lytic infection, our data reveal some new aspects of their functions in promoting lytic replication. Unlike previous studies suggesting that RNA1.2 and RNA2.7 do not affect lytic growth kinetics^13,14^, we found that 1.2-Mut and 2.7-Mut showed slight but significant reductions in virus production compared to that of the WT. The main reason for this may be the titration period. Previous studies examined viral growth after saturation, but our growth kinetics data showed that RNA1.2 and RNA2.7 accelerated lytic replication when viral growth is unsaturated. Notably, we revealed that virus production of 4.9-Mut was dramatically decreased than that of the WT during lytic replication. A previous study reported the role of RNA4.9 which is dependent on R-loop formation in the *oriLyt* region^12^, and our results showed that not only *oriLyt,* but the 3′ region of RNA4.9, play crucial roles in promoting lytic replication. Moreover, it remains unclear if RNA5.0 regulates viral growth kinetics during lytic replication, despite not being essential for viral production^24^. We confirmed that 5.0-Mut showed no defects in viral growth kinetics in fibroblasts, indicating that RNA5.0 does not promote lytic replication.

Myeloid lineage cells latently infected with HCMV exhibit restricted viral gene expression. However, several latency-associated viral proteins, such as UL138 and LUNA, is required for viral latency and reactivation^29,30^. Based on our results, we present viral lncRNAs as novel viral factors in maintaining latent reservoirs. Furthermore, we observed that the lncRNA-mediated maintenance of latent reservoirs was essential for efficient viral reactivation, which is closely related to severe clinical symptoms. Interestingly, 2.7-Mut showed the most deleterious phenotype in maintaining latent reservoirs, which is distinct from the result that 4.9-Mut showed the greatest fold reduction in progeny virus production during lytic infection. These data indicated that HCMV lncRNAs have specific functions in latency distinguished from those in lytic replication. Generally, persistent DNA viruses tether their viral episome to host chromatin and replicate their DNA to maintain viral reservoirs during latency^76,77^. Considering the nuclear localization of RNA4.9 within the VRC, lncRNA-mediated maintenance of HCMV reservoirs could depend on tethering of the viral episome to host chromatin. Alternatively, considering the anti-apoptotic function of RNA2.7^13,78^, lncRNAs may sustain latent reservoirs by inhibiting cell death. Future studies are needed to clarify the detailed mechanism by which these three lncRNAs differentially maintain latent viral reservoirs.

Nanopore DRS has been widely utilized to characterize the epitranscriptomic features of viral transcripts^79,80^. Comparing DRS data from native RNA with that of the IVT negative control revealed 36 putative modified sites of RNA1.2, RNA2.7, and RNA4.9, containing m^6^A consensus motif “AGACH” (Fig. 4c and d). In addition to the previous data revealing m^6^A modification of RNA4.9 in lytic infection^81^, our nanopore DRS and MeRIP data suggested that RNA1.2, RNA2.7, and RNA4.9 are modified with m^6^A throughout the entire viral life cycle. Furthermore, we found that the m^6^A readers, YTHDF2 and IGF2BP3, interacted considerably with the three lncRNAs in all infection states and promoted lncRNA stability (Fig. 5c and d). Although IGF2BP3 is a well-known RNA-stabilizing protein, YTHDF2 is generally thought to degrade RNA by deadenylation^73,74^. However, the RNA-stabilizing function of YTHDF2 has been reported^82^, supporting our results that YTHDF2 stabilizes HCMV lncRNAs. Moreover, YTHDF2 exhibits pro-viral activity on HCMV lytic infection by targeting interferon transcripts^81^. Our data additionally suggest the pro-viral function of YTHDF2 by stabilization of HCMV lncRNAs. Altogether, our global epitranscriptomic dissection discovered HCMV exploits m^6^A modification to maintain high expression levels of viral lncRNAs which are essential throughout viral life cycle.

Aside from m^6^A modification, our molecular analyses provide ground dataset to understand the function of HCMV lncRNAs. Our DRS of the HCMV transcriptome throughout the entire viral life cycle identified differentially modified regions of HCMV transcripts in each infection state, revealing broadly reduced RNA modifications in latency compared with those seen in reactivation or lytic infection (Fig. 4a and b). Dynamic reprogramming of epitranscriptome observed in cell differentiation or in life cycle of other viruses, such as Karposi’s sarcoma-associated herpesvirus, enables the specific regulation of RNA in each biological context^21,37,83^. Because RNA1.2, RNA2.7, and RNA4.9 are the predominant targets of differential RNA modification (Fig. 4b), the functions of the three lncRNAs can be distinguished by differential RNA modifications across the viral life cycle. Furthermore, DRS data showed 20 out of 36 modified sites contain m^6^A motif (Fig. 4c), implying that the remaining 16 sites may represent other types of RNA modifications. Considering that N^1^-methyladenosine (m^1^A) or 5-methylcytosine (m^5^C) modifications, are also frequently observed in viral transcripts and required for viral pathogenesis^84,85^, other RNA modifications in the remaining sites might have critical roles in HCMV lncRNA function.

Moreover, modified RAP-MS data identified unique binding proteins for each lncRNA that have specific functions (Fig. 5a). In particular, the long poly(A) tail and high stability of RNA2.7 may be engaged by RNA2.7-unique binding proteins that promote RNA stability. Furthermore, considering that the function of DNA-binding lncRNAs, such as X-inactive specific transcript (XIST), depends on their interaction with nuclear proteins^86^, RNA4.9 could regulate viral DNA integrity via interacting with its unique binding proteins that localized in the nucleus. These unique binding protein subsets will provide clues as to how each lncRNA is specifically involved in host-virus interactions. We also revealed that a substantial portion of common binding proteins contained antiviral RBPs (Fig. 5b). The antiviral activity of RBPs is triggered not only by RNA viruses, but also by RNAs transcribed from DNA viruses^87^. In light of the abundant expression of these three lncRNAs, innate immune responses during HCMV infection may be tightly regulated in a viral lncRNA-dependent manner. Consequently, these datasets could be utilized as ground information to provide insights into the functions of HCMV lncRNAs.

Currently used HCMV drugs mostly target viral proteins such as viral DNA polymerase or terminase complex to restrict virus propagation in lytic replication. However, mounting evidences showed the limitation of these drugs including toxicity and rapid emergence of drug resistance^88^. Furthermore, recently developed therapeutics target viral latency and reactivation^89,90^, which requires finding new targets throughout the whole viral life cycle for HCMV therapeutics. In the present study, we suggest that three HCMV lncRNAs, RNA1.2, RNA2.7, and RNA4.9, are potential targets of HCMV therapeutics in all infection states. Moreover, our comprehensive molecular analysis of HCMV lncRNAs revealed how they interact with host RNA-processing machineries, and provided extensive dataset to elucidate their mechanisms of action. We expect this study will contribute to understanding viral lncRNA-mediated host-virus interaction.

## Methods

### Cell culture

HFFs (SCRC-1041; American Tissue Culture Collection [ATCC]) were grown in Dulbecco’s modified Eagle’s medium (DMEM; Hyclone) with 10% fetal bovine serum (FBS; HyClone), GlutaMAX-I (Gibco), and penicillin (100 U/mL)-streptomycin (100 μg/mL) (Gibco). Kasumi-3 cells (CRL-2725; ATCC) were cultured in Roswell Park Memorial Institute medium (RPMI 1640; ATCC) supplemented with 20% FBS, penicillin–streptomycin, and 100 μg/mL gentamicin (Sigma). Cells were incubated at 37 °C in a 5% CO_2_ atmosphere.

### Viral infection

HCMV particles were generated by the transfection of HCMV Toledo BAC DNA (Gifts from T. Shenk; Princeton University) into primary HFFs via electroporation using the Neon Transfection System (MPK10096; Invitrogen) following manufacturer instructions. After 100% cytopathy was observed, culture media containing viral particles were collected and centrifuged at 40,000 × *g* for 40 min at 4 °C, and virus pellets were resuspended in serum-free medium. For titration of viral stocks, HFFs were infected with serially diluted viruses for 1 h and fixed with 3.7% formaldehyde at 24 h post-infection. Cells were then permeabilized with 0.1% Triton X-100 and incubated with blocking solution (2% BSA in PBS) and stained with HCMV IE1 antibody (MAB810R; Millipore) and FITC-conjugated anti-mouse antibody (115–095-146; Jackson Laboratories). Cells were then mounted with DAPI-containing solution (H-1200; Vector Laboratory). The number of HCMV IE1–positive cells was counted, and the MOI was calculated by the ratio of IE1-positive to total cells.

For lytic HCMV infection, HFFs were incubated with the virus in serum-free DMEM for 1 h, washed twice with PBS, and incubated in complete media. For latent HCMV infection and subsequent reactivation, Kasumi-3 cells were incubated in X-VIVO 15 (Lonza) for 48 h before infection. Cells were infected at an HCMV multiplicity of infection (MOI) of 1 by incubation in X-VIVO 15 for 4 h with rocking every 30 min, followed by spinfection at 1000 × *g* for 30 min. After overnight incubation with the virus, cells were treated with trypsin–EDTA to detach the remaining viral particles, and then cushioned onto Ficoll-Pacque (GE Healthcare Life Sciences) to separate live cells from debris and viruses. The cells over the cushion were washed with PBS and incubated in X-VIVO 15. For the reactivation assay, cells were treated with 20 nM phorbol 12-myristate 13-acetate (PMA) or DMSO for an additional 2 days. Kasumi-3 cells were seeded at a two-fold serial dilution from 1600 to 13 cells per well in a 96-well plate containing primary HFF cells and cultured for 14 d. The frequency of infectious centers was calculated using an ELDA^91^.

### Generation of lncRNA-Mut virus

HCMV lncRNA-Mut was constructed from HCMV Toledo BAC (GenBank AC146905.1) using a counter-selection BAC modification kit (Gene Bridge), according to manufacturer protocol. Briefly, a target region in the Toledo BAC DNA was replaced with a prokaryotic selectable marker, *rpsL-*neo^R^, by homologous recombination. PCR products with flanking sequences homologous to the target regions were introduced by electroporation (1652660; Bio-Rad). BAC clones with an *rpsL-*neo^R^ cassette were selected using kanamycin and verified by PCR. For RNA1.2 and RNA2.7, the lncRNA sequences were completely replaced with the cassette. RNA4.9 sequences, excluding *oriLyt*, were also replaced with the cassette. For RNA5.0, the cassette was replaced with sequences harboring modified splice donor sites (AG-GT → CT-CG). The modified regions were replaced with the original lncRNA sequences and successful clones were selected using streptomycin. The primers used for BAC recombination are listed in Supplementary Table 5. PCR- and sequencing-verified BAC clones were extracted using the NucleoBond Xtra BAC kit (740436; Macherey–Nagel). Recombinant BACs were transfected into primary HFFs for propagation, as described in “Viral infection”.

### Northern blot analysis

To generate antisense RNA probes targeting lncRNAs, ~ 200 nt of RNA containing T7 were synthesized by PCR. RNA probes were synthesized by in vitro transcription (IVT) of α-^32^P UTP using the EZ T7 High Yield In Vitro Transcription kit (EZ027S; Enzynomics) according to manufacturer instructions. Briefly, approximately 1 μg of template was incubated in transcription mixture (0.5 mM each ATP, CTP, and GTP; 1 mCi/mL α-^32^P UTP; 1 mM DTT; 20 units of RNase inhibitor [M007; Enzynomics]; 1× transcription buffer; and 200 units of T7 RNA polymerase) for 6 h at 37 °C. Template DNA was then digested using recombinant DNase I (2270; Takara) at 37 °C for 1 h, followed by inactivation via supplementation with 1 μL of 0.5 M EDTA at 80 °C for 2 min. Finally, the probes were purified using a NucleoSpin RNA Clean-up Kit (740948; Macherey–Nagel) and resuspended in nuclease-free distilled water. The primers used for the production of IVT templates are listed in Supplementary Table 6.

Primary HFFs in 100 mm dishes (approximately 2 × 10^6^ cells) were infected with HCMV at an MOI of 3. At 3 d post-infection, total RNA was extracted using TRIzol reagent (15596026; Invitrogen), according to manufacturer instructions. For the removal of genomic DNA, total RNA was treated with recombinant DNase I (2270; Takara) for 1 h at 37 °C, then purified using a NucleoSpin RNA Clean-up kit (740948; Macherey–Nagel). Northern blotting was performed using the NorthernMax kit (AM1940; Invitrogen) according to manufacturer protocol. Briefly, 10 μg purified RNA was dissolved in formaldehyde. RNA samples were then separated in 1% formaldehyde agarose gels with Millennium RNA markers (AM7150; Invitrogen) in MOPS gel running buffer. After electrophoresis, the lane containing the size marker was excised and stained separately with EtBr. The remaining samples in the MOPS gel were transferred to BrightStar-Plus membranes (AM10104; Invitrogen) via capillary transfer for 4 h at 25 °C. Membranes were UV cross-linked (120 mJ/cm^2^) and preincubated in UltraHyb hybridization buffer for 30 min at 68 °C. Then, α-^32^P UTP-labelled northern probes were added to the membrane and incubated overnight at 68 °C with constant rotation. The following day, the membranes were washed twice with a low-stringency wash solution and twice with a high-stringency wash solution. The blots were exposed to Fuji 32P screens and scanned using Typhoon FLA7000 (ver 1.2).

### Immunoblot analysis

Cells were lysed in RIPA buffer (50 mM Tris [pH 7.4], 150 mM sodium chloride, 0.5% sodium deoxycholate, 0.1% SDS, and 1.0% NP-40) supplemented with 10 μM leupeptin (L8511; Sigma-Aldrich) and 1 mM phenylmethanesulfonylfluoride (P7626; Sigma-Aldrich). Prior to SDS-PAGE, samples were boiled in SDS sample buffer and separated on 10% gels. The samples were then transferred onto nitrocellulose membranes (10-401-196; Whatman). After transfer, membranes were blocked with 5% skim milk in Tris-buffered saline with 0.1% Tween-20 (TBST) for 1 h and incubated with primary antibodies at 4 °C. After overnight incubation, the membranes were washed three times with TBST and incubated with peroxidase-conjugated secondary antibodies diluted to 1:5000 in 5% skim milk with TBST. After washing three times with TBST, signals were detected using a SuperSignal West Pico chemiluminescent substrate (34580; Thermo Fisher Scientific). The primary antibodies used were IE1/2 (MAB810R; Millipore), UL44 (CA006-1; Virusys), pp28 (CA004-1; Virusys), and GAPDH (AbFrontier). Peroxidase-conjugated anti-mouse IgG (115-035-062) and anti-rabbit IgG (111-035-003; Jackson Laboratories) were used as secondary antibodies.

### Measurement of viral RNA and DNA via RT-PCR and qPCR

Total RNA from HCMV-infected cells was extracted using TRIzol reagent (15596026; Invitrogen), according to manufacturer instructions. To remove genomic DNA, total RNA was treated with recombinant DNase I (2270; Takara) for 1 h at 37 °C, then purified using a NucleoSpin RNA Clean-up kit (740948; Macherey–Nagel). cDNA was synthesized using the ReverTra Ace qPCR RT Kit (FSQ-201; Toyobo), according to the manufacturer protocol. Genomic and viral DNA were purified using the QIAamp DNA Blood Mini Kit (Qiagen) following manufacturer instructions. Real-time PCR was performed using TOPreal qPCR 2× PreMIX (RT500; Enzynomics). Viral RNA levels were normalized against GAPDH expression. The qPCR primers used in the present study are listed in Supplementary Table 7.

### smFISH and IFA

We designed a set of probes for each HCMV lncRNA using the Stellaris Probe Designer with a masking level of 5. The probes were divided into equimolar mixtures of odd and even sets. A previously described method was used to produce labeled FISH probes, with minor modifications^92^. Briefly, probes were labeled with Cy5-ddCTP (NU-850-CY5; Jena Bioscience) in 15 μL of TdT reaction mixture (0.8 U/μL terminal deoxynucleotidyl transferase; EP0161; Thermo Fisher Scientific), 1 nmol oligo mixture, 1× TdT reaction buffer, and 5 nmol Cy5-ddCTP) overnight at 37 °C. The following day, the Cy5-probe mixture was precipitated with ethanol and resuspended in nuclease-free distilled water. The sequences of the FISH probes are listed in Supplementary Table 8.

HFF cells were seeded on an 18 mm coverslip in 12-well plates and cultured to 60–70% confluency. After HCMV infection, cells were fixed with 3.7% formaldehyde in PBS for 10 min, then quenched with 0.1 M glycine in PBS for 10 min. Cells then were permeabilized by adding 70% ethanol for 3 h to 7 days at 4 °C. Before FISH staining, cells were rehydrated with PBS for 30 min and incubated in pre-hybridization solution (2× SSC with 10% formamide) for 30 min at 37 °C. Hybridization was performed in 50 μL of hybridization solution (2× SSC, 10% formamide, 10% dextran sulfate, 50 μg yeast tRNA, 0.2% BSA, 50 units of RNase inhibitor (Enzynomics), and 50 ng Cy5-labeld probes) overnight at 37 °C. The next day, the cells were washed twice with pre-hybridization buffer for 30 min. For the immunofluorescence experiment, the hybridized cells were incubated in a blocking solution (10% formamide, 2× SSC, and 0.2% BSA) for 1 h, then incubated with anti-IE1 primary antibody (MAB810R, Millipore) diluted to 1:200 in blocking solution for 2 h. The cells were washed twice with the pre-hybridization solution for 15 min and incubated with FITC-conjugated anti-mouse secondary antibody (115-095-146; Jackson Laboratories) diluted in blocking solution (1:200) for 1 h. The cells were washed twice more as described above, and coverslips were mounted on glass slides using Vectashield antifade medium with DAPI (H-1200; Vector Laboratories). Images were obtained using Nikon Eclipse Ti2 inverted microscope equipped with a 1.45 numerical aperture (NA) Plan apo λ 100× oil objective and an sCMOS camera (Photometrics prime 95 B 25 mm). For each field of view, images were taken with DAPI395, GFP488, and Atto647 channels using the NIS-Elements software.

### IVT of HCMV lncRNAs

To generate template DNAs for IVT, we designed a sense primer with T7 promoter (5′-TAATACGACTCACTATAGGG-3′) and an antisense primer with 20 nt of T, which produced transcribed RNA with 20 nt of poly(A) stretch. To reduce non-specific amplification from complex or long lncRNAs, RNA4.9 and RNA5.0 templates, respectively, for IVT were generated by two successive rounds of PCR. In the first, templates containing lncRNA sequences were amplified from HCMV Toledo BAC DNA using “Nested PCR primer”. Next, templates for IVT of HCMV lncRNAs were amplified from the first PCR DNA using “IVT primer,” followed by gel extraction of the desired band. Template DNA was treated with proteinase K (200 μg/mL) and 0.5% SDS for 30 min at 50 °C, extracted with equal volumes of phenol/chloroform/isoamyl alcohol, and precipitated with ethanol. The primers used for the production of IVT templates are listed in Supplementary Table 9.

IVT was performed using the MEGAscript T7 kit (AM1334; Invitrogen) according to manufacturer protocol, with several modifications. Briefly, 500 ng of template DNA was incubated with 20 μL of IVT mixture (30 mM NTP mixture, 40 units of RNase inhibitor (Enzynomics), 1X reaction buffer, and 2 μL T7 enzyme mix) overnight at 37 °C. The next day, transcribed RNAs were treated with 1 μL TURBO DNase for 15 min at 37 °C, followed by precipitation with 30 μL LiCl precipitation solution and resuspension in nuclease-free DW. The resulting suspension was used for subsequent nanopore library construction.

### Illumina total RNA sequencing

For Illumina sequencing, a total of 500 ng RNA per sample from latent (8 days post-infection) or reactivated (8 + 2 days post-infection with PMA) virus was used. The NGS library was constructed using the TruSeq Stranded Total RNA Library LT Sample Preparation kit with Ribo-Zero Gold (RS-122-2301; Illumina) according to manufacturer instructions. The libraries were sequenced on a NovaSeq 6000 system (Illumina) in paired-end mode to a length of 2 × 100 bp. The sequenced libraries were aligned to the reference sequences in the nanopore DRS using STAR^93^. Transcript quantifications were performed with “featureCount” in the subread package^94^.

### Nanopore direct RNA sequencing (DRS)

For nanopore DRS, total RNA was extracted using TRIzol reagent (15596026; Invitrogen), according to manufacturer instructions. Total RNA was then treated with recombinant DNase I (2270; Takara) for 1 h at 37 °C, followed by purification using a NucleoSpin RNA Clean-up kit (740948; Macherey–Nagel). Purified total RNA or 500 ng of RNA from IVT was prepared using a direct RNA sequencing kit (SQK-RNA002, Oxford Nanopore Technologies) with a modification to include 20 U of SUPERase-In RNase inhibitor (Ambion, 20 U/uL) in adapter ligation reactions. The prepared libraries were loaded onto R9.4.1 flow cells (FLO-MIN106D, Oxford Nanopore Technologies) and sequenced using MinION Mk1b devices (MinKNOW 4.1.2, Oxford Nanopore Technologies).

The nanopore direct RNA sequencing data were basecalled using guppy 4.2, an RNA research flip-flop model (res_rna2_r941_min_flipflop_v001, available from the Rerio repository of Oxford Nanopore Technologies). The sequence reads were aligned to the reference sequence database comprising the human genome (GENCODE Human Release 36), the virus genome (GU937742.2), yeast ENO2 cDNA (SGD YHR174W), and human ribosomal DNA complete repeat unit (GenBank U13369.1) using minimap2 (version 2.17^95^) with options “-k 13 -x splice -N 32 -un”. The reads were also aligned to fully-spliced transcript sequences comprising the human transcriptome (GENCODE human release 36), the virus transcriptome (GenBank GU937742.2), yeast ENO2 cDNA (SGD YHR174W), and human ribosomal DNA complete repeat unit (GenBank U13369.1) using minimap2 with options “-Ktg -uf -ax splice”. For differently expressed gene analysis, expression was quantified using “featureCount” in the subread package^94^ with option “—primary -L”. Sequences mapped to the viral genome were extracted and re-aligned to the viral genome or transcriptome using minimap2 options “-k 8 -w 1 –splice -g 30000 -G 30000 -A1 -B2 -O2,24 -E1,0 -C0 -z 400,200 –no-end-flt –junc-bonus=50 -N 32 –splice-flank=no –max-chian-skip=40 -un –MD -a -p 0.7” to improve the alignment quality. lncRNA reads were extracted using bedtools 2.28 “intersect” and subsampled for further analysis^96^. The length of the poly(A) tails was measured using Nanopolish 0.13.2 [65]. For the coverage depth plot, viral reads were aligned to the viral genome and sequencing read coverage was calculated using the “genomecov” command in bedtools 2.28. The coverage depths were binned to 30-nucleotide bins and plotted using medians.

### Splicing analysis of viral RNA

For coverage and % spliced of transcripts, reads mapped to transcripts were selected from transcriptome aligned file. Coverage of selected reads were computed per genome base, and long deletion was caculated using python lab manual. Shortly, cigar string was extracted and skipping region (N) longer than 40 nt was considered as long deletion. Long deletion per total coverage per genome base were calculated as % spliced.

### Detection of modified bases and statistical analysis

For overall modification analysis, 3 lncRNAs and 22 viral genes with high expressions (> 100 reads in all samples) were selected from transcriptome alignment. To calculated relative modificiation rate between two samples in base resolution, error rates (%ESB) were compared with the other samples in every permutation using ELIGOS2 pair_diff_mode. The resultant combine files of all comparisons were merged, and the sites which exceeded threshold, minimap coverage (> 50), minimum odds ratio (> 2), maximum adjustment p-value (< 0.001) were considered as possible differentially modified sites. Those candidates were bineed to 6 nucleotide bins and summarized by using maximum odds ratio*.*

For HCMV lncRNA modification analysis, the DRS data of HCMV lncRNAs and IVT RNAs were downsampled so that the each results contains roughly less than 4000 coverage. Downsampled RNA reads were realigned to the virus genome using minimap2 with options “-k 6 w 1” to improve sensitivity. Modified base detection was done by ELIGOS2. Downsampled reads were calculated its error rated and those were compared for a basecalling error analysis using ELIGOS2 “pair_diff_mod” model.

The confident set of modified sites was selected based on the following criteria: minimum coverage (> 100), minimum odds ratio (> 1.8), and maximum adjusted p-value (< 0.001). The candidate modification sites were binned to 4-nucleotide bins and summarized using the maximum odds ratio. The motif searches near the modification sites were performed using the MEME suite 5.3.0 with the “-objfunc cd -mod zoops -minw 5” option^97^.

### MeRIP

Total RNA was extracted from HCMV-infected HFF (lytic infection), Kasumi-3 (latent infection), and PMA-treated Kasumi-3 (reactivation) cells. Total RNA was treated with recombinant DNase I (Takara) for 1 h at 37 °C and purified using TRIzol LS (Invitrogen). Poly(A) RNA enrichment was performed with 150 μg of total RNA using a Poly(A) Purist Mag kit (Invitrogen), followed by ethanol precipitation. Eluted RNA was incubated with 1.5 μg of anti-m^6^A antibody (Merck, ABE572) in MeRIP buffer (50 mM Tris, pH 7.5, 150 mM NaCl, 1 mM EDTA, and 0.1% NP-40) for 2 h at 4 °C. The immunoprecipitation mixture was mixed with 1 mg of Dynabead protein A (Invitrogen) and incubated overnight at 4 °C. Beads were then washed five times with MeRIP buffer, and RNA was eluted twice by incubation in MeRIP buffer containing 6.7 mM m^6^A sodium salt (Sigma) for 1 h at 4 °C. Eluted and bead-bound RNA were purified using the NucleoSpin RNA Clean-up kit (Macherey–Nagel) and subjected to RT-PCR.

### Preparation of biotinylated probes

Biotinylated probes were prepared in a manner similar to that previously described^42^. Briefly, to purify the RNP complex of HCMV lncRNAs, we generated biotinylated antisense oligonucleotide (ASO) probes targeting the sense strand of each lncRNA based on the reference HCMV Toledo genome (GenBank accession GU937742.2). The template for ASOs targeting sense-lncRNAs was designed with 90 nt oligomers and densely overlapping adjacent oligomers at 60 nt (Supplementary Table 3). To avoid non-specific RNAs, we discarded oligomers aligned to the human transcriptome (as of Oct 14, 2019) using Bowtie 2^98^. Reverse complement oligomers for each ASO template were also designed to generate antisense lncRNA-targeting probes. To amplify the ASO template, sequence elements for IVT, reverse transcription (RT), and PCR were added to the ASO templates. The T7 promoter (5′-TAA TAC GAC TCA CTA TAG GG-3′) and pad for RT priming (5′-TGG AAT TCT CGG GTG CCA AGG-3′) were added to the head and tail of each tile, respectively. The final ASO templates (167 nt) were prepared via oligo pool synthesis by GenScript. The ASO target sequences used in the present study are listed in Supplementary Table 3.

Amplification of ASO templates was performed using KAPA HiFi HotSart ReadyMix (Roche) and PCR primers for each ASO pool. The PCR products were purified using a QIAquick PCR Purification Kit (QIAGEN). IVT was performed using the MEGAscript T7 transcription kit (Invitrogen), and the DNA templates were degraded using TURBO DNase (Invitrogen). To purify transcribed RNA, a 1.8X reaction volume of AMPure XP (Beckman) was applied, polyethylene glycol was added to a final concentration of 20%, and size selection was performed according to manufacturer protocol. Biotinylated ASOs were generated using RevertAid Reverse Transcriptase (Thermo Scientific) and 5′ biotin-TEG primers. RNA intermediates were hydrolyzed with 0.1 M NaOH and neutralized with acetic acid. The final ASO purification was carried out in the same manner as IVT RNA purification. The primer sequences used for PCR and RT are listed in Supplementary Table 3.

### Modified RAP-MS

Modified RAP was performed in a similar manner in the previous study^42^, with some variations. Per replicate for each lncRNA, 60 million HFFs were infected with HCMV Toledo (3 MOI) and detached from culture dishes by trypsin. The cell pellets were resuspended with 6 mL of ice-cold PBS and dispersed in 100 mm dishes to irradiate 254 nm UV for 800 mJ/cm^2^ using Spectrolinker XL-1500. UV-crosslinked cells were frozen with liquid phase of nitrogen and stored at − 80 °C. Frozen cell pellet was thawed and resuspended in TURBO DNase solution (10 Units per million cells) and incubated at 37 °C for 1 h. DNA digested cells were supplemented with equal volume of pre-heated 2X lysis buffer (40 mM Tris–HCl at pH 7.5, 1 M LiCl, 1% LDS, 2 mM EDTA, 10 mM DTT and 8 M urea) and denatured by incubating at 68 °C for 30 min. Lysate was centrifuged at 15,000 g for 20 min to remove insoluble pellet. Take out 1/100th of “Input RNA” sample and store it at 4 °C to measure RNA yield later. Remaining cell lysates were loaded into Amicon 100 K filter (Merck) that had been previously washed with 1X lysis buffer. Spin down cell lysates at 3200 g and 4 °C until upper chamber volume reduces to 1/7th of original volume. UV-crosslinked RNP complexes were concentrated in the upper chamber. Concentrated RNP was split into three of 2 mL protein-LOBIND tube (Eppendorf) and each sample was mixed with 6 μg biotin probe pools (Each sense-lncRNA or antisense-lncRNA targeting probes) or distilled water (No probe). Hybridization was performed by incubating at 68 °C for 10 min and rotation at room-temperature for 3 h. Biotin-labeled RNP lysates were supplemented with streptavidin beads (300 μL per sample, New England Biolabs) and captured by rotating at room temperature overnight. Probe-enriched RNP beads were washed with 1X lysis buffer and transferred to fresh tubes, followed by final wash with detergent-free wash buffer (20 mM Tris–HCl at pH 7.5, 0.5 M LiCl, 1 mM EDTA). 1/10^th^ of beads were set aside for assessment of RNA contents by RT-qPCR. The remaining 9/10th of beads were digested with 100 units of Benzonase nuclease (Millipore) at 37 °C for 1 h. 1/10th of digested samples were used for silver staining (KOMA biotech) and the remaining 9/10^th^ of digested samples were subjected for MS analysis. For on-bead peptide digestion, nuclease treated beads were suspended to final 8 M urea and reduced with 10 mM dithiothreitol (DTT), alkylated with 40 mM iodoacetamide (IAA) for 1 h each at 37 °C, and diluted with 50 mM ammonium bicarbonate (ABC) to final 1 M urea. These bead suspensions were supplemented with 300 ng Trypsin (Thermo Scientific, MS grade) and 1 mM CaCl_2_ and digested overnight at 37 °C. Peptide solutions were separated from magnetic beads and subjected with HiPPR detergent removal spin columns (Thermo Scientific) and desalted by reverse phase C18 ziptip (Millipore). After the clean-up, the samples were reconstituted with 20 μL of 25 mM ammonium bicarbonate buffer for LC–MS/MS analysis.

To measure RNA yield, input RNA sample and Probe-enriched RNA samples were digested with 100 μg of Proteinase K (Thermo) at 50 °C for 40 min and boiled at 95 °C for 30 min to reverse cross-linking. The samples were diluted with distilled water to 250 μL and mixed with 750 μL of TRIzol LS (Invitrogen). Total RNA was extracted as described above, and yield of RNA1.2, RNA2.7 and RNA4.9 was measured by RT-qPCR. For silver staining, Benzonase eluted samples were transferred to fresh tube and mixed with Laemmli sample buffer (Biorad). For PAGE analysis, protein samples were separated in Novex WedgeWell 10–20% Tris–Glycine Mini Gel (Invitrogen) and the gel was fixed with 10% acetic acid and 40% Ethanol solution and processed with silver staining (KOMA biotech) by manufacturer’s recommendation.

### LC–MS/MS analysis

LC–MS/MS analysis was performed using an Orbitrap Fusion Lumos Tribrid MS (Thermo Fisher Scientific) coupled with a nanoAcquity UPLC system (Waters). Both the analytical capillary columns (100 cm × 75 μm i.d.) and trap columns (3 cm × 150 μm i.d.) were packed with 3 μm Jupiter C18 particles (Phenomenex, Torrance). The long analytical column was placed in a column heater (Analytical Sales and Services) regulated to a 45 °C. The LC flow rate was 300 nL/min, and the 100 min linear gradient ranged from 95% solvent A (H_2_O with 0.1% formic acid (Merck)) to 40% solvent B (100% acetonitrile with 0.1% formic acid). Precursor ions were acquired at a 120 K resolving power of m/z 200, and precursor isolation for MS/MS analysis was carried out at 1.4 Th. Higher-energy collisional dissociation with 30% collision energy was used for sequencing, with a target value of 1 × 10^5^ ions determined by automatic gain control. The resolving power for the acquired MS2 spectra was set to 30 K at m/z 200, with a maximum injection time of 120 ms. Mass spectrometry raw data files were processed for label-free quantification using MaxQuant (version 2.0.3.0).

Statistical analysis was conducted in a manner similar to that previously described^42^. Briefly, data from the "no probe" and "antisense probe" experiments were used to account for various technical factors. The “no probe” experiments controlled for technical interactors, such as biotin-containing carboxylases. The "antisense probe" experiments represented possible RNA-binding proteins retrieved from non-specific RNA targets. Utilizing these two technical backgrounds, we identified a list of proteins that were specifically enriched in the target experiment and thus represented candidate HCMV lncRNA regulators. The two technical replicates were aggregated and Laplace's rule of succession was used to handle zero and missing values. The protein spectral count data were modelled as a multinomial distribution. Finally, the Benjamini–Hochberg method was used to control the false discovery rate (FDR).

GO terms for significant binding proteins of the three lncRNAs were analyzed by over-representation analysis using WebGestalt (WEB-based GEne SeT AnaLysis Toolkit). All proteins detected by modified RAP-MS were used as reference gene sets. The minimum and maximum numbers of genes for each category were designated as 5 and 2000, respectively.

### RNA immunoprecipitation

For lytic infection lysates, HFF cells were infected with HCMV at an MOI of 3 and harvested after 3 days. For lysates of reactivated latent samples, Kasumi-3 cells were infected at an MOI of 1, as previously described. Latency samples were harvested at 8 days post-infection, and reactivation samples were incubated for an additional 2 days with 20 nM PMA before harvesting. A total of 6 × 10^6^ HFF cells or 5 × 10^6^ Kasumi-3 cells were collected per sample. Cells were resuspended in RIP lysis buffer (150 mM KCl, 10 mM HEPES [pH 7.6], 2 mM EDTA, 0.5% NP40, 1 mM DTT, 10 μM leupeptin, 1 mM phenylmethanesulfonylfluoride, and 400 unit/mL RNase inhibitor [Enzynomics]) for 10 min at 4 °C. Lysates were centrifuged at 15,000 × *g* for 15 min at 4 °C, then filtered by passing through a 0.44 μm membrane syringe filter. Respective input samples for RNA extraction and immunoblot assays were saved (10% of lysates). For antibody-bead preparation, 2 μg YTHDF2 (24744-1-AP; Proteintech), IGF2BP3 (RN009P; MBL Life Science), or normal rabbit IgG (sc-2027; Santa Cruz Biotechnology) antibodies were diluted in NT2 buffer (200 mM NaCl, 50 mM HEPES [pH 7.6], 2 mM EDTA, and 0.05% NP40) and incubated with Dynabead protein G (Invitrogen) for 1 h with rotation at 25 °C. After incubation, the antibody-bead complexes were washed and resuspended in an equivalent volume of lysate. Lysates were then added to the antibody-bead mixture and incubated overnight on a rotating wheel at 4 °C. The following day, the beads were washed five times with ice-cold NT2 buffer. The 10% reserves of beads were resuspended in RIP lysis buffer and analyzed by immunoblotting with input samples. The remaining 90% of beads and input RNA samples were mixed with 1 mL of TRIzol (Invitrogen) and subjected to RNA extraction.

### Stability of HCMV lncRNAs in m^6^A machinery–depleted cells

Initially, 5 × 10^4^ HFF cells were seeded in 24-well plates and transfected with gene-specific siRNA using Dharmafect-1 reagent (Dharmacon) at a final concentration of 30 nM. At 72 h after transfection, the cells were infected with HCMV at an MOI of 1. One hour after infection, another set of siRNA was transfected. At 72 h after infection, cells were incubated with 5 μM actinomycin D and harvested for RNA extraction.

To capture nascent RNA, we used the Click-iT Nascent RNA Capture Kit (C10365; Invitrogen), according to manufacturer instructions. Briefly, 72 h after infection, cells were incubated in 20 nM EU-containing medium for 2 h. Three h after labeling, the cells were harvested for RNA extraction. Total RNA was extracted using TRIzol reagent, and genomic DNA was digested using recombinant DNase I (Takara) at 37 °C for 1 h. A 500 ng sample of total RNA was then biotinylated in reaction cocktail (1× Click-iT EU buffer, 2 mM CuSO_4_, 0.25 mM biotin azide, 10 mM Click-iT reaction buffer additive 1, and 12 mM Click-iT reaction buffer additive 2) for 1 h in a vortex mixer at 25 °C. Biotinylated RNA was precipitated using ethanol and resuspended in RNase-free water. To capture biotinylated RNAs, an equivalent volume of 2× Click-iT RNA binding buffer was added to the biotinylated RNA. The RNA binding reaction mix was heated at 70 °C for 5 min and immediately mixed with 12 μL Dynabead MyOne Streptavidin T1 (Invitrogen) per reaction. The bead-RNA mix was incubated for 30 min in a vortex mixer at 25 °C. The beads were then washed five times with wash buffers 1 and 2. After the final wash, beads were resuspended in 14 μL of wash buffer 2 and cDNA synthesis was performed immediately using the ReverTra Ace qPCR RT Kit (Toyobo).

## Supplementary Information

Supplementary Information 1.

Supplementary Information 2.

Supplementary Information 3.

## Data Availability

All sequencing data generated for this study are deposited in the NCBI Gene Expression Omnibus (GEO) database (GSE217714). Associated code is available at https://github.com/ChangLabSNU/hcmv_lnc_modi. The mass spectrometry proteomics data have been deposited to the ProteomeXchange Consortium via the PRIDE^99^ partner repository with the dataset identifier PXD034587 and 10.6019/PXD034587.
