# Supplementary figures and images for: Functional and molecular dissection of HCMV long non-coding RNAs
Source: Sci Rep. 2022 Nov 11;12:19303. doi: 10.1038/s41598-022-23317-3 (PMC9652368; doi:10.1038/s41598-022-23317-3)

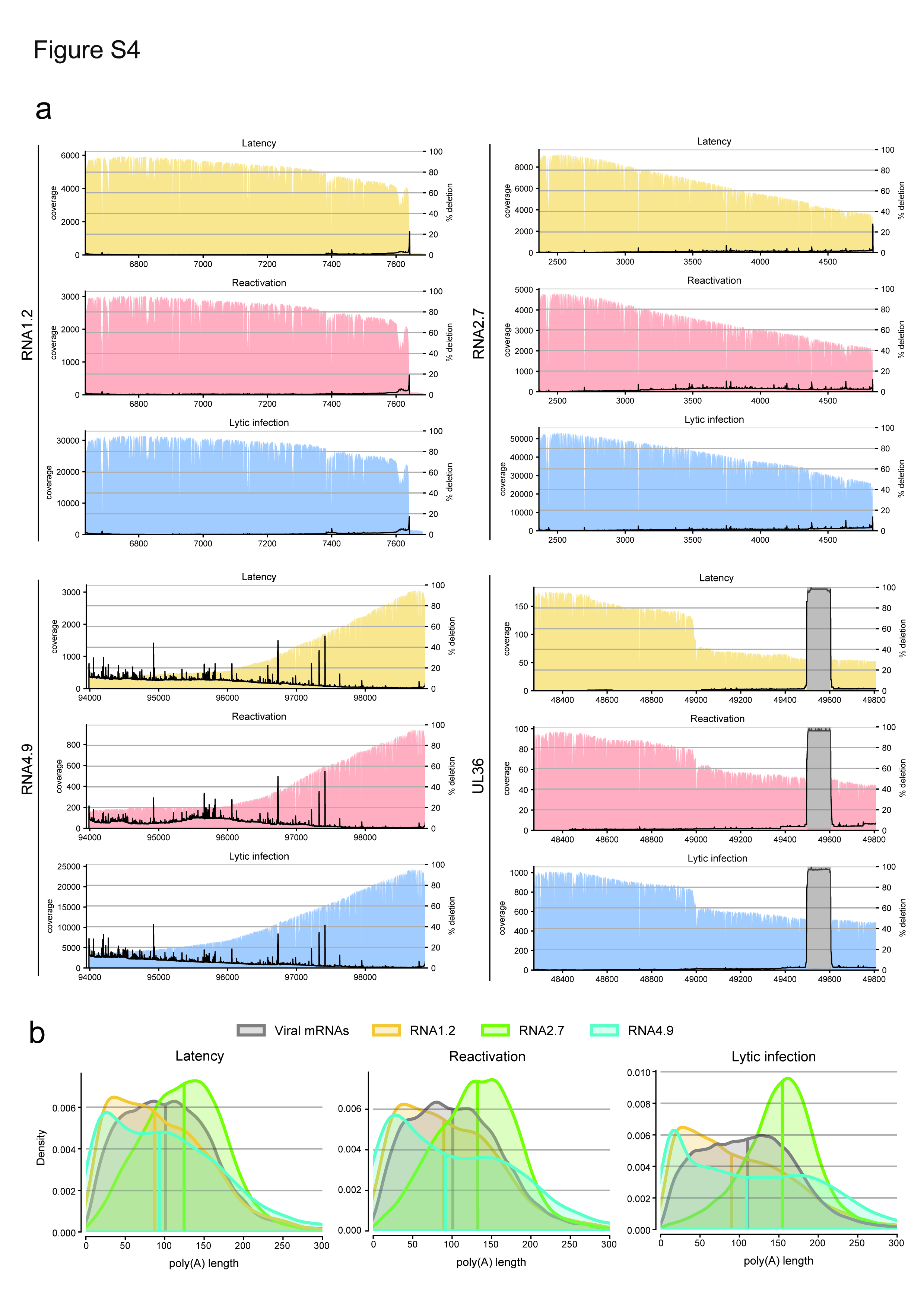

Supplement: Supplementary file 2 — Supplementary Information 2. [file 41598_2022_23317_MOESM2_ESM.zip › Supplementary Figure 4.tif]

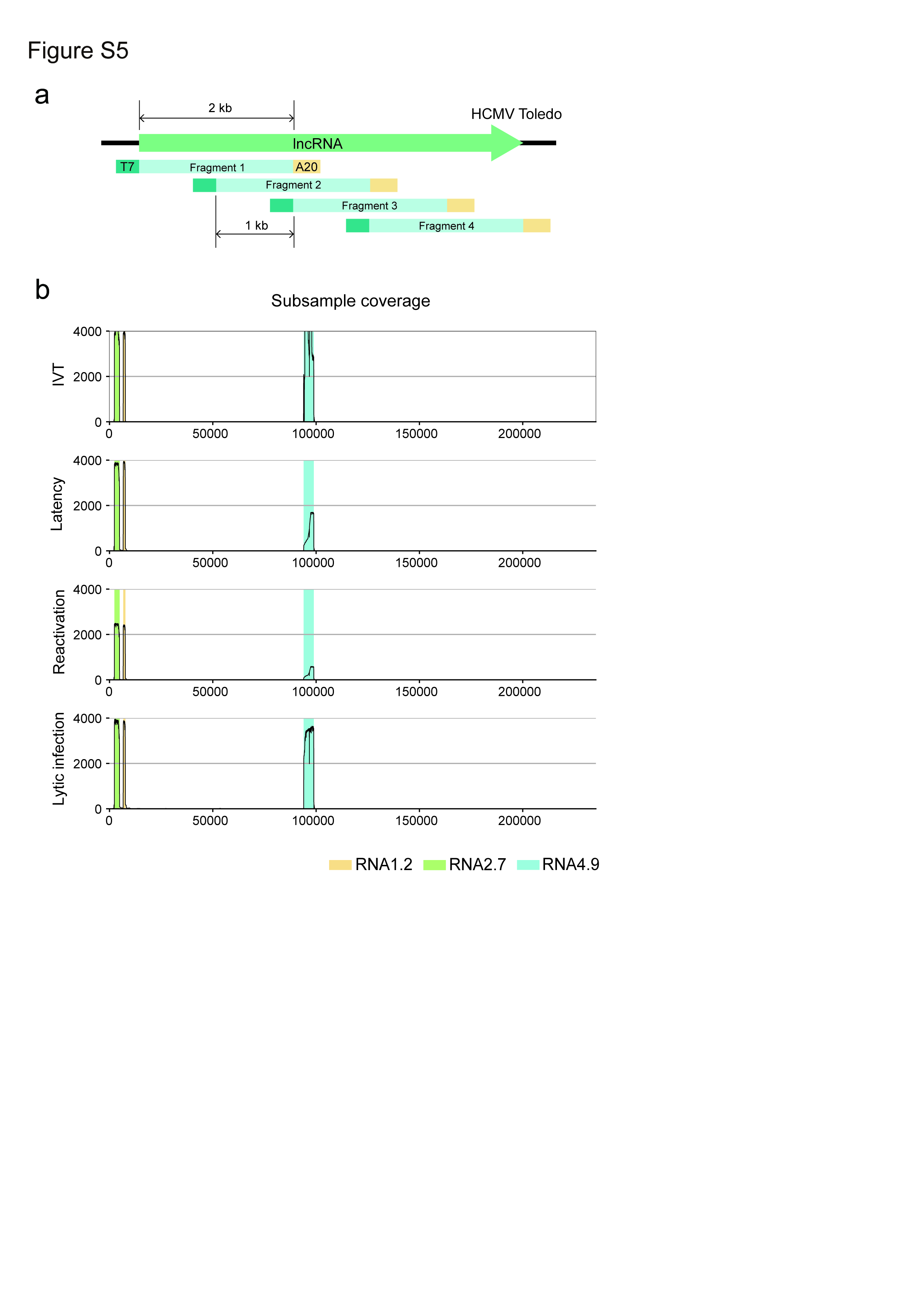

Supplement: Supplementary file 2 — Supplementary Information 2. [file 41598_2022_23317_MOESM2_ESM.zip › Supplementary Figure 5.tif]

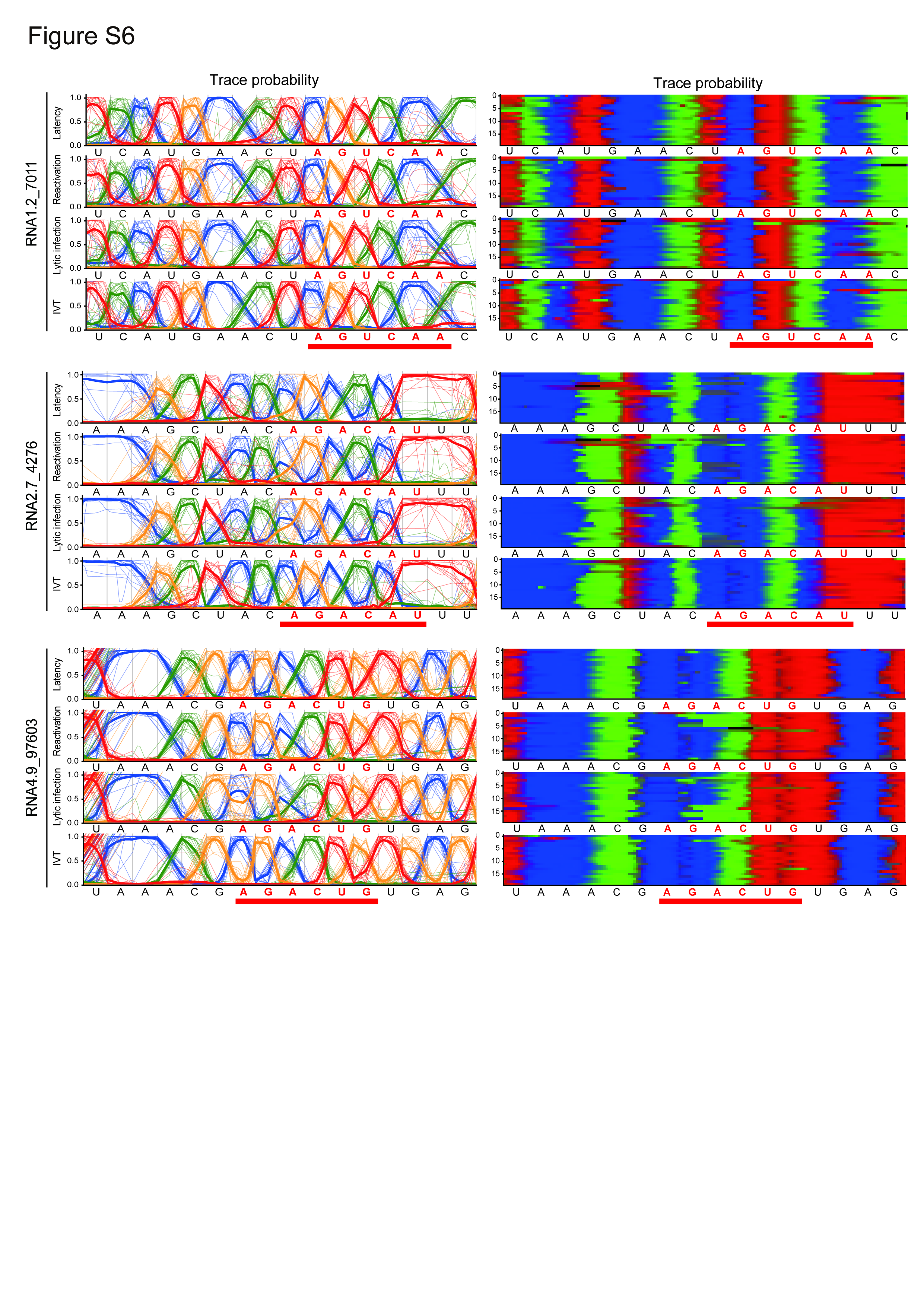

Supplement: Supplementary file 2 — Supplementary Information 2. [file 41598_2022_23317_MOESM2_ESM.zip › Supplementary Figure 6.tif]

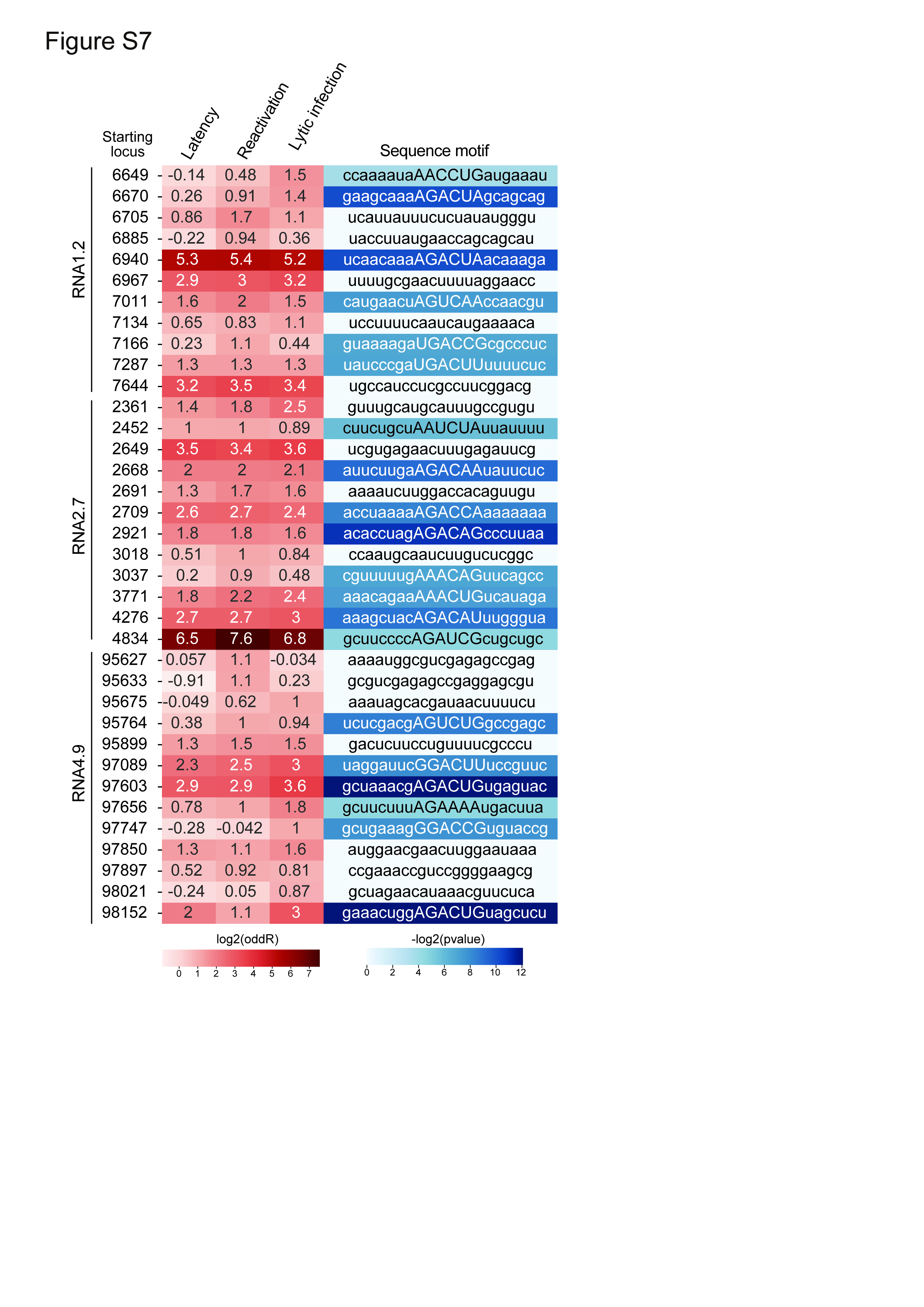

Supplement: Supplementary file 2 — Supplementary Information 2. [file 41598_2022_23317_MOESM2_ESM.zip › Supplementary Figure 7.tif]

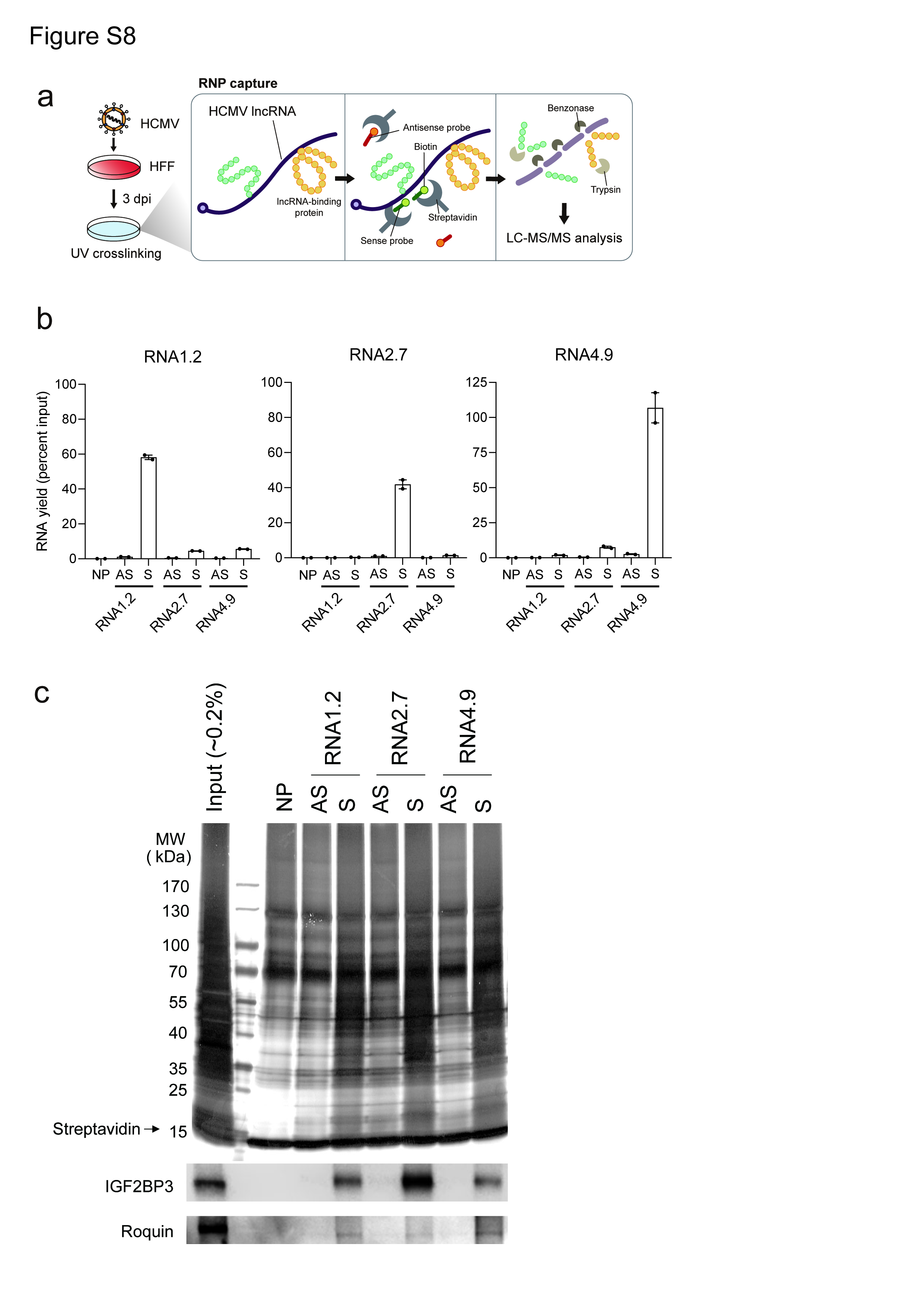

Supplement: Supplementary file 2 — Supplementary Information 2. [file 41598_2022_23317_MOESM2_ESM.zip › Supplementary Figure 8.tif]

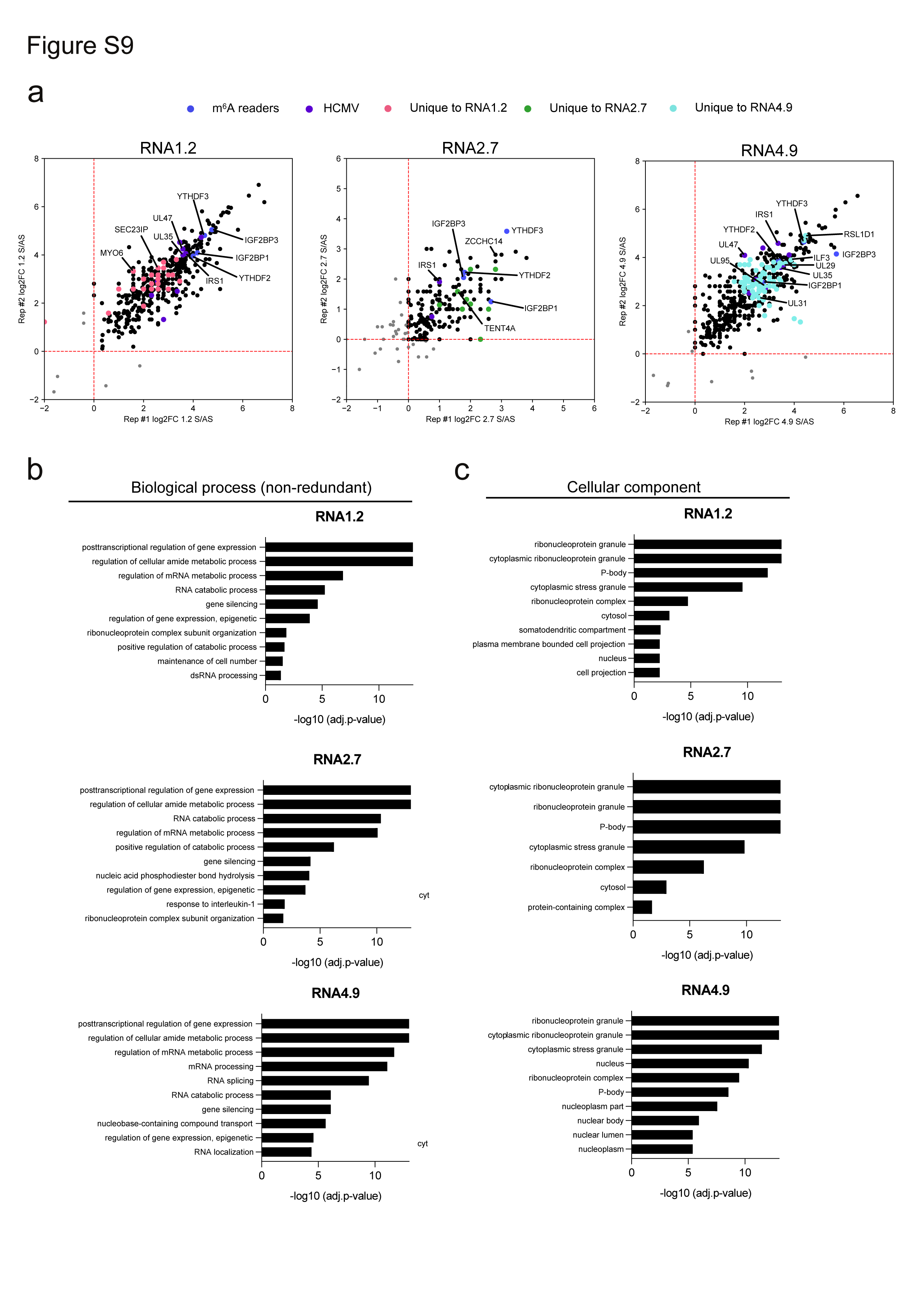

Supplement: Supplementary file 2 — Supplementary Information 2. [file 41598_2022_23317_MOESM2_ESM.zip › Supplementary Figure 9.tif]

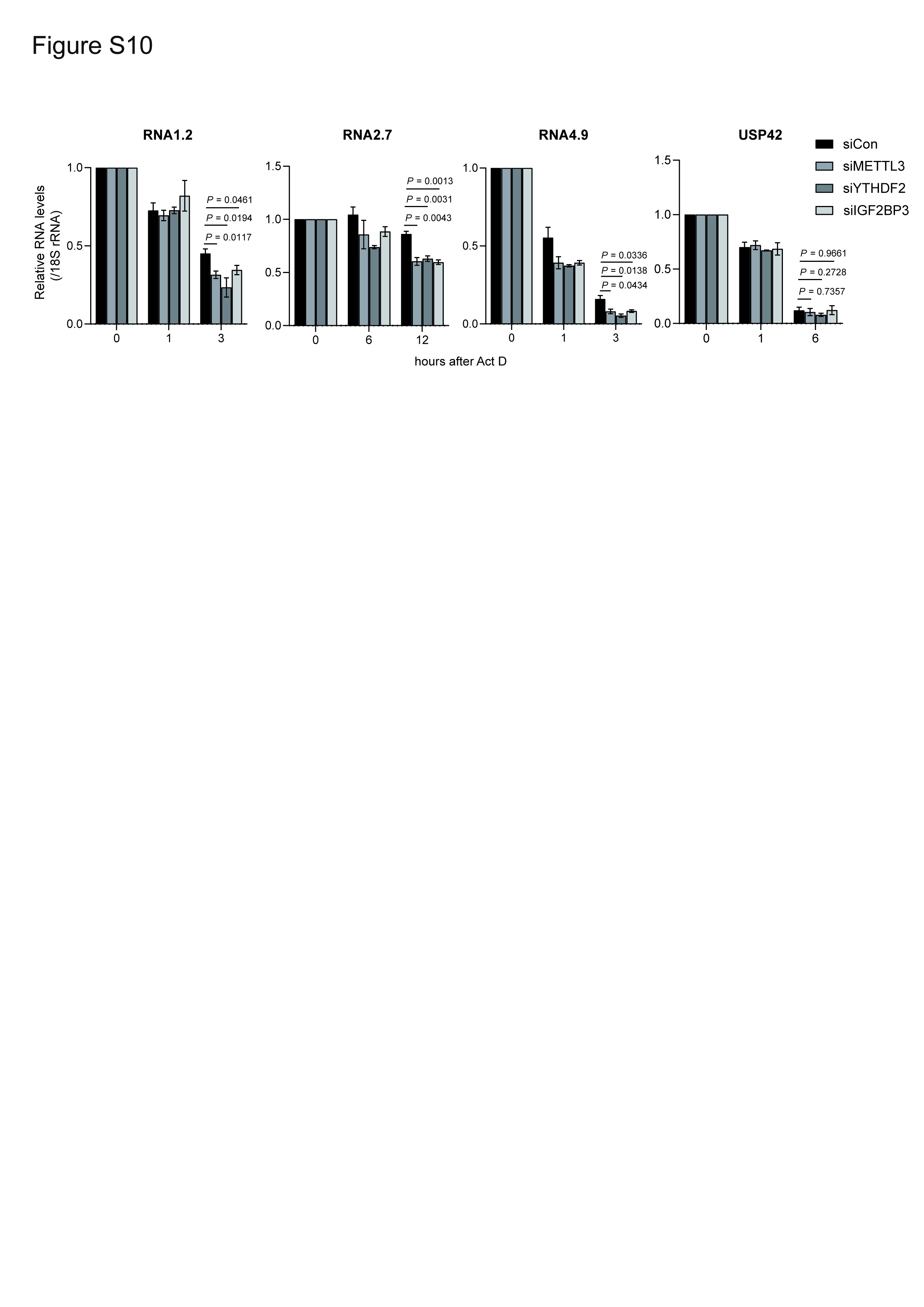

Supplement: Supplementary file 2 — Supplementary Information 2. [file 41598_2022_23317_MOESM2_ESM.zip › Supplementary Figure 10.tif]

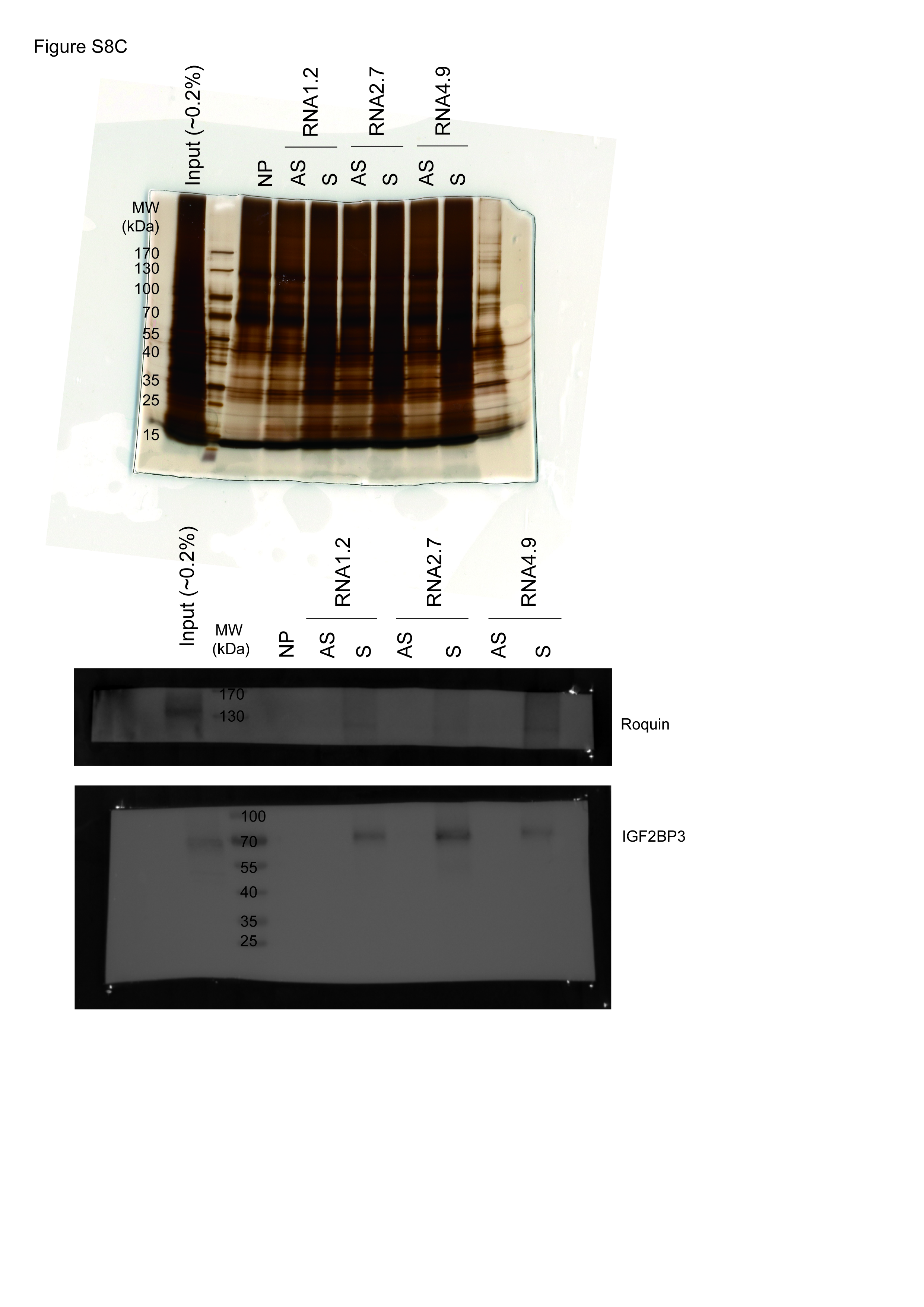

Supplement: Supplementary file 2 — Supplementary Information 2. [file 41598_2022_23317_MOESM2_ESM.zip › Source data-Figre S8C.tif]

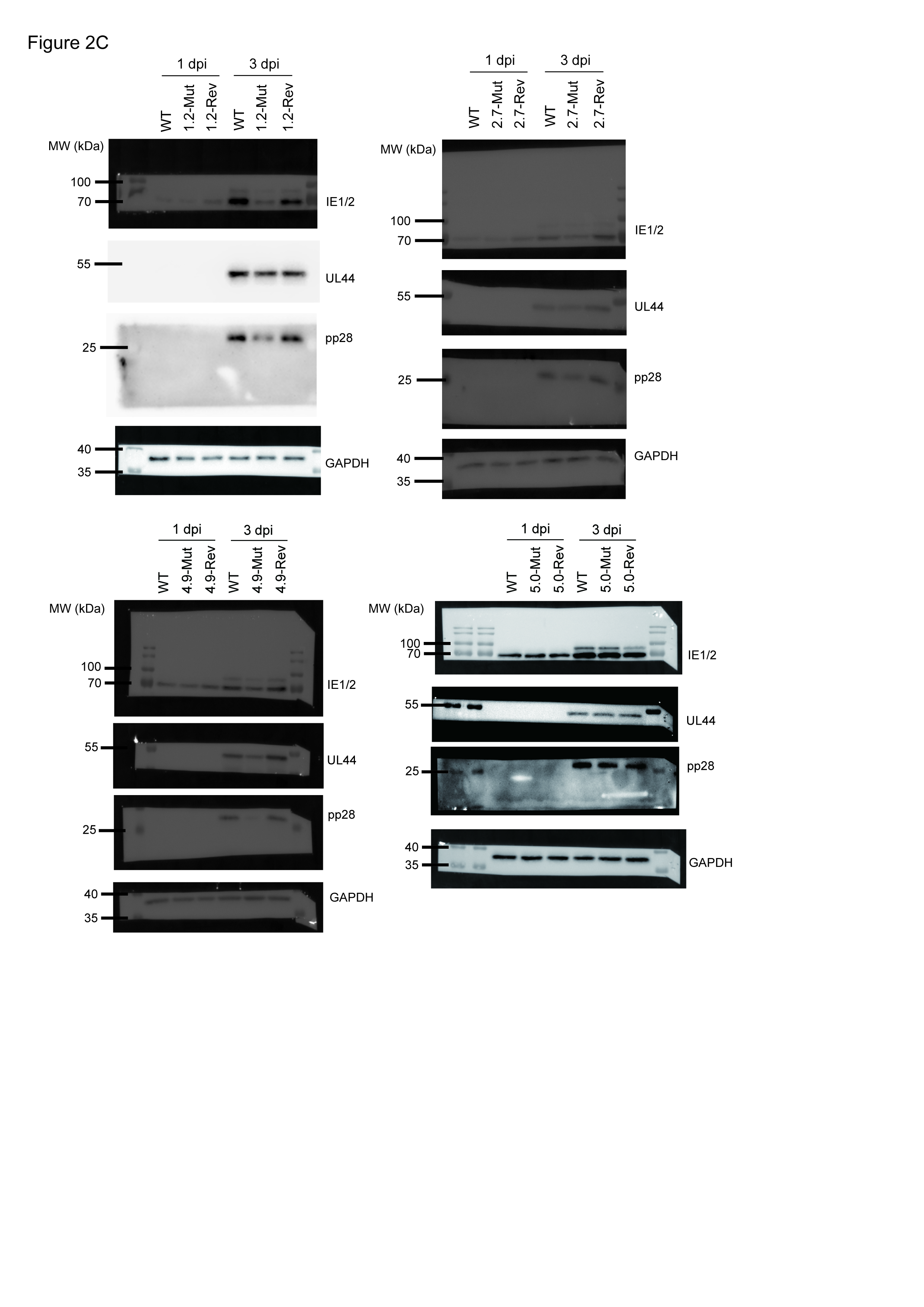

Supplement: Supplementary file 2 — Supplementary Information 2. [file 41598_2022_23317_MOESM2_ESM.zip › Source data-Figure 2C.tif]

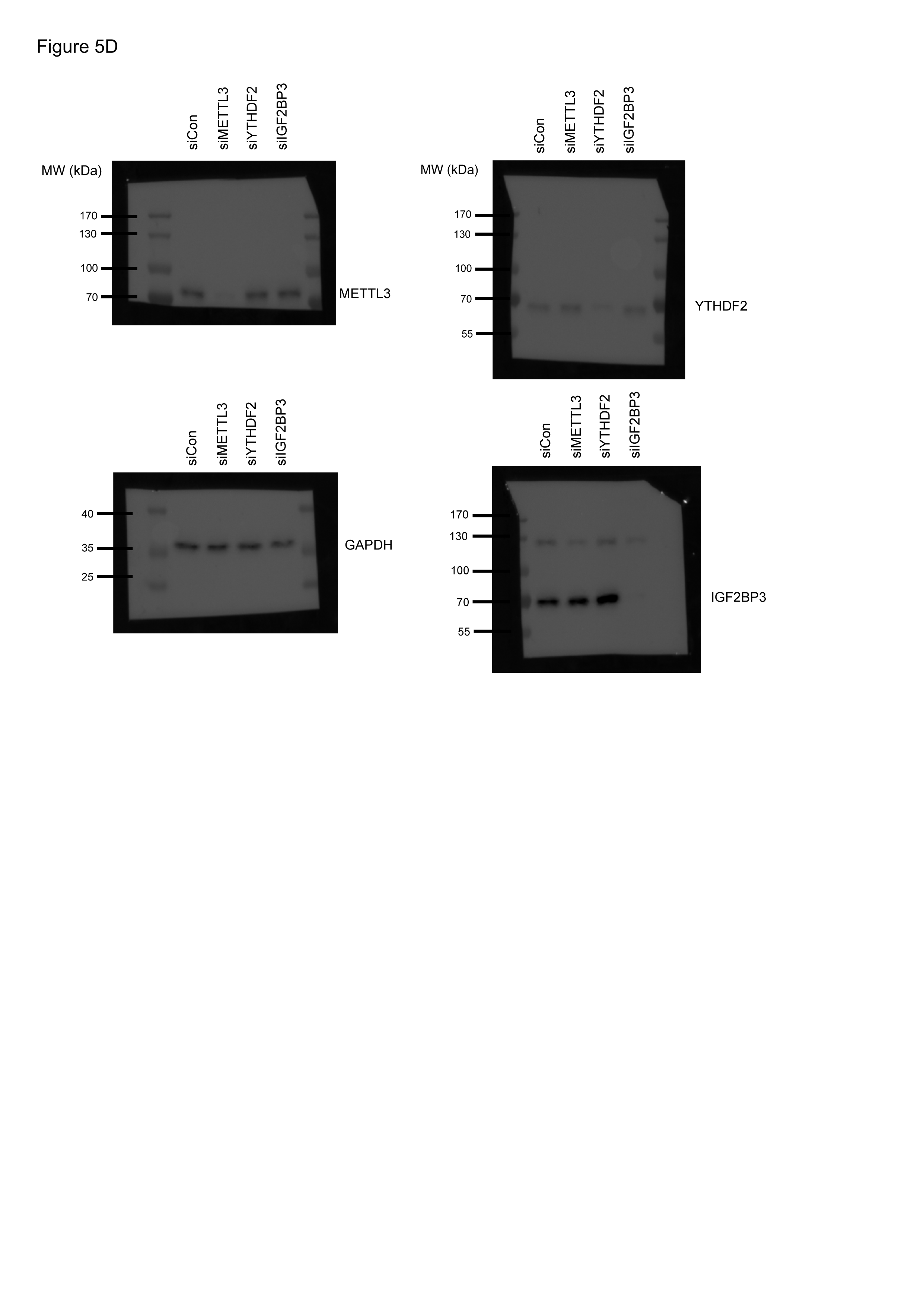

Supplement: Supplementary file 2 — Supplementary Information 2. [file 41598_2022_23317_MOESM2_ESM.zip › Source data-Figure 6D.tif]

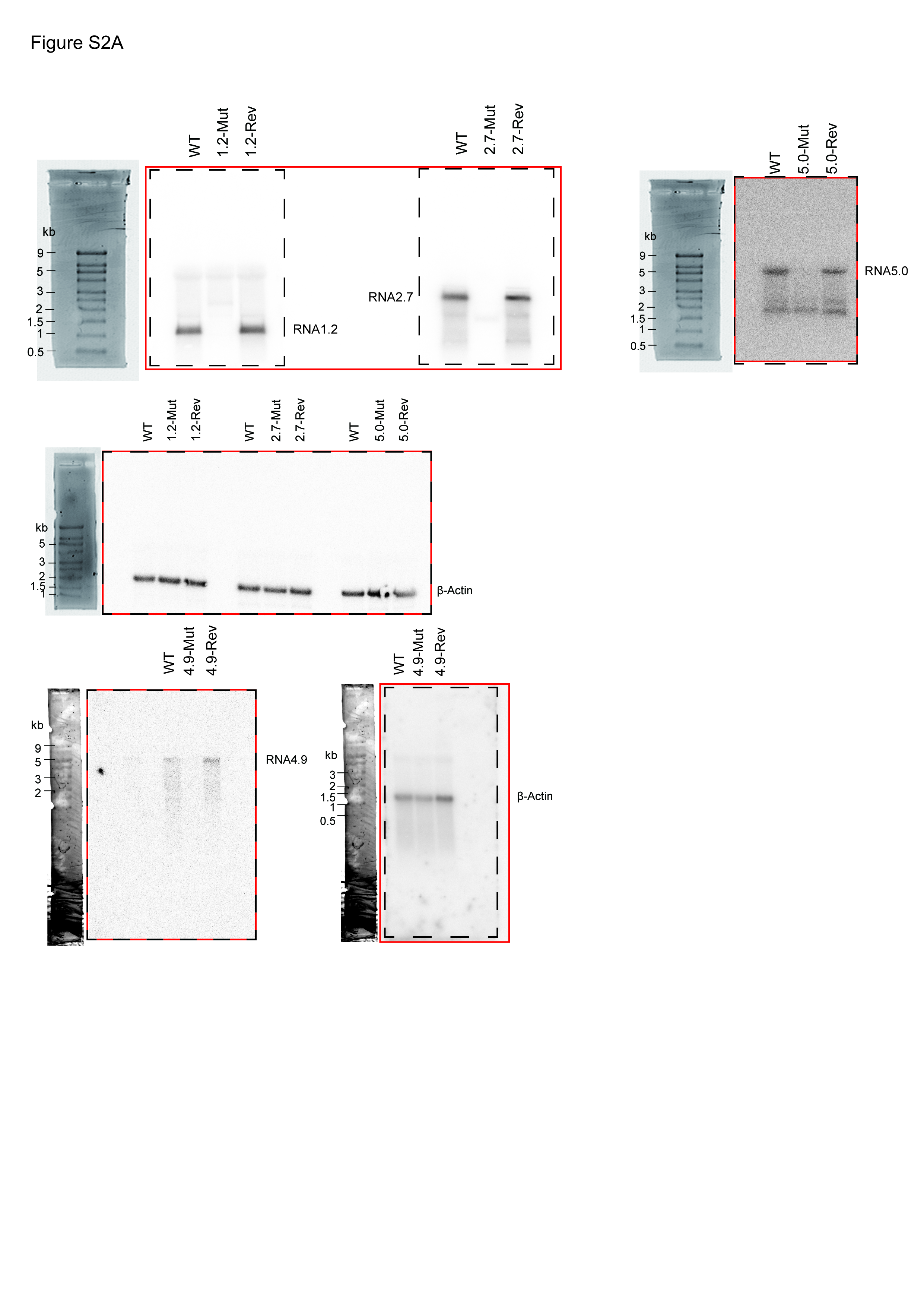

Supplement: Supplementary file 2 — Supplementary Information 2. [file 41598_2022_23317_MOESM2_ESM.zip › Source data-Figure S2A.tif]

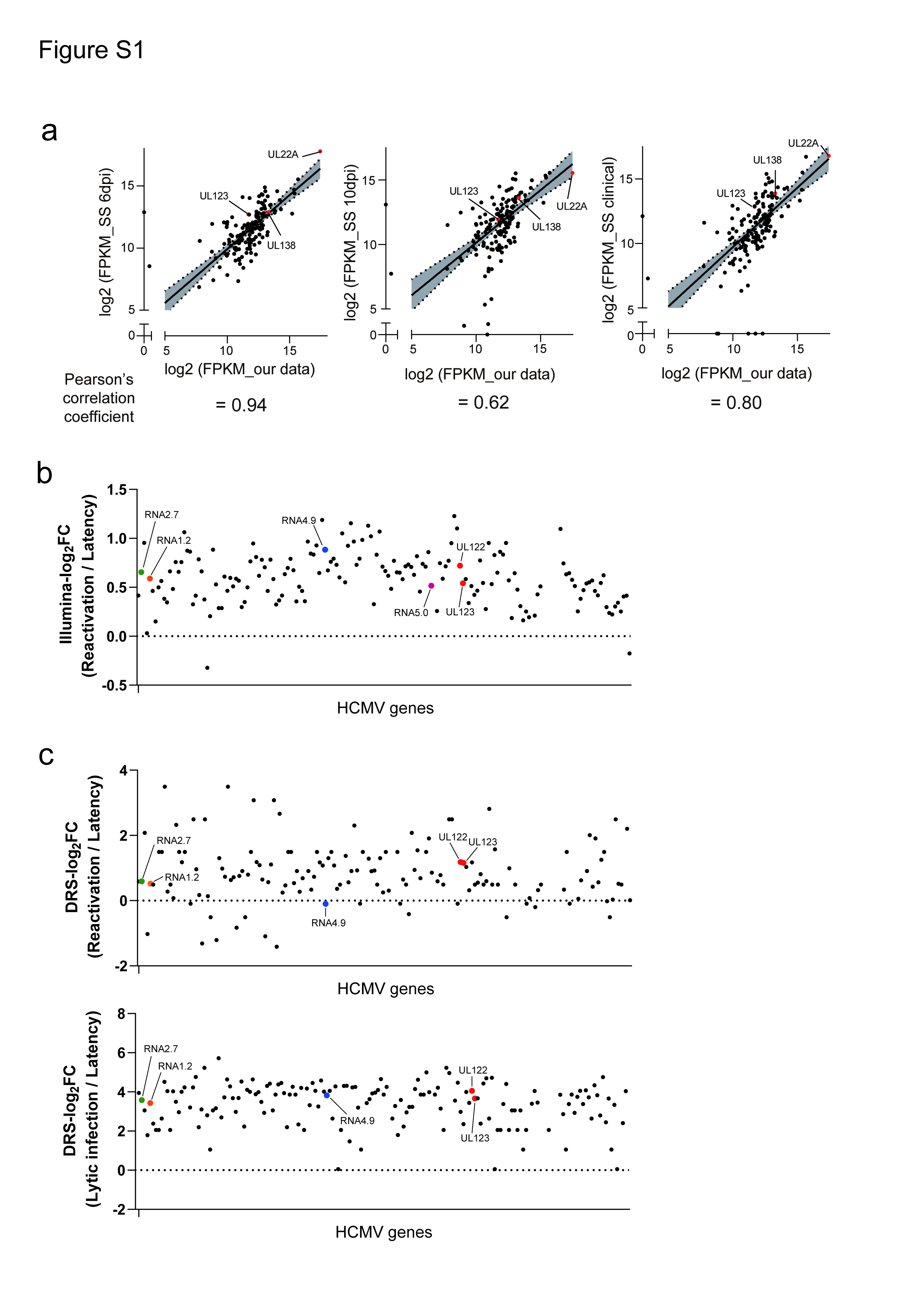

Supplement: Supplementary file 2 — Supplementary Information 2. [file 41598_2022_23317_MOESM2_ESM.zip › Supplementary Figure 1.tif]

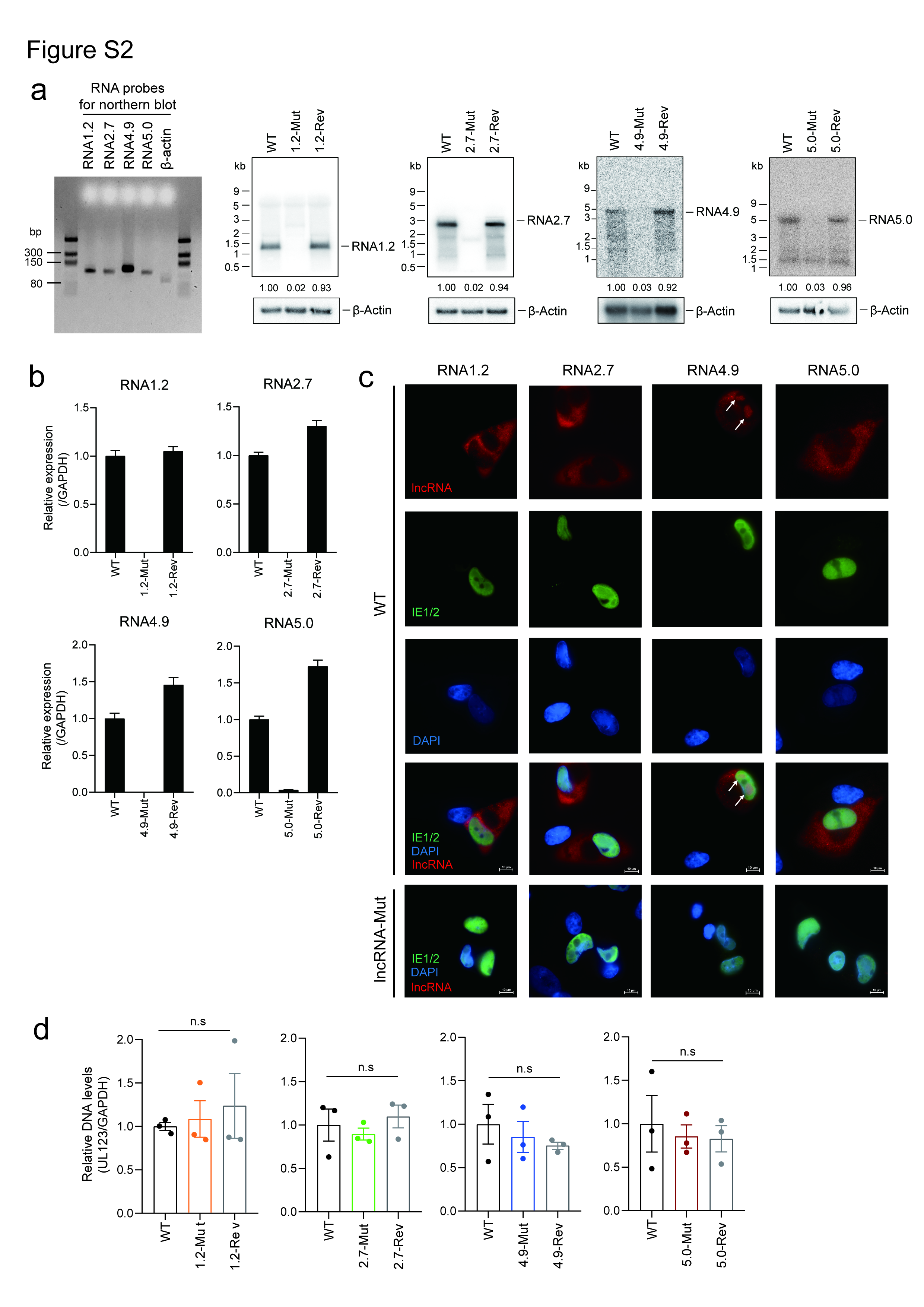

Supplement: Supplementary file 2 — Supplementary Information 2. [file 41598_2022_23317_MOESM2_ESM.zip › Supplementary Figure 2.tif]

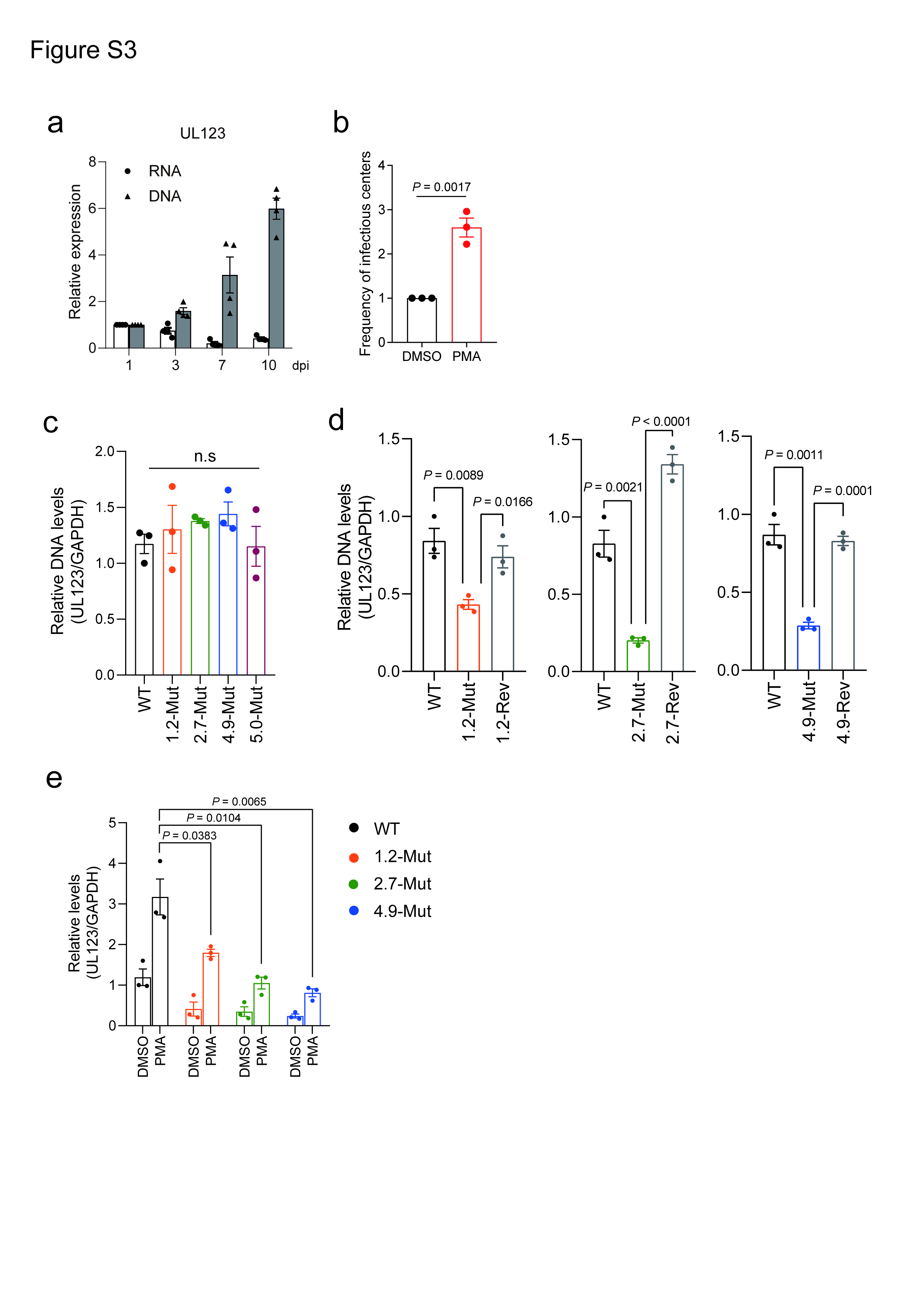

Supplement: Supplementary file 2 — Supplementary Information 2. [file 41598_2022_23317_MOESM2_ESM.zip › Supplementary Figure 3.tif]
