# Supplementary material for: Functional and molecular dissection of HCMV long non-coding RNAs
Source: Sci Rep. 2022 Nov 11;12:19303. doi: 10.1038/s41598-022-23317-3 (PMC9652368; doi:10.1038/s41598-022-23317-3)

SUPPLEMENTARY INFORMATION FOR

Functional and molecular dissection of HCMV long non-coding RNAs

Sungwon Lee,[1,2†] Hyewon Kim,[1,2†] Ari Hong,[2,3†] Jaewon Song,[1,2†] Sungyul Lee,[1,2] Myeonghwan Kim,[1,2] Sung-yeon Hwang,[1,2] Dongjoon Jeong,[1,2] Jeesoo Kim,[1,2] Ahyeon Son,[1,2] Young-suk Lee[4], V. Narry Kim,[1,2] Jong-seo Kim,[1,2] Hyeshik Chang,[1,2,3*] and Kwangseog Ahn[1,2*]

[1]School of Biological Sciences, Seoul National University, Seoul 08826, Republic of Korea

[2]Center for RNA Research, Institute for Basic Science, Seoul 08826, Republic of Korea

[3]Interdisciplinary Program in Bioinformatics, Seoul National University, Seoul 08826, Republic of Korea

[4]Department of Bio and Brain Engineering, Korea Advanced Institute of Science and Technology (KAIST), Daejeon 34141, Republic of Korea

*Corresponding authors (hyeshik@snu.ac.kr, ksahn@snu.ac.kr)

[†]These authors contributed equally to this work.

This file includes

Supplementary Fig. 1 to 10

Source data

Other supplementary materials for this manuscript include the following :

Supplementary Table 1-9

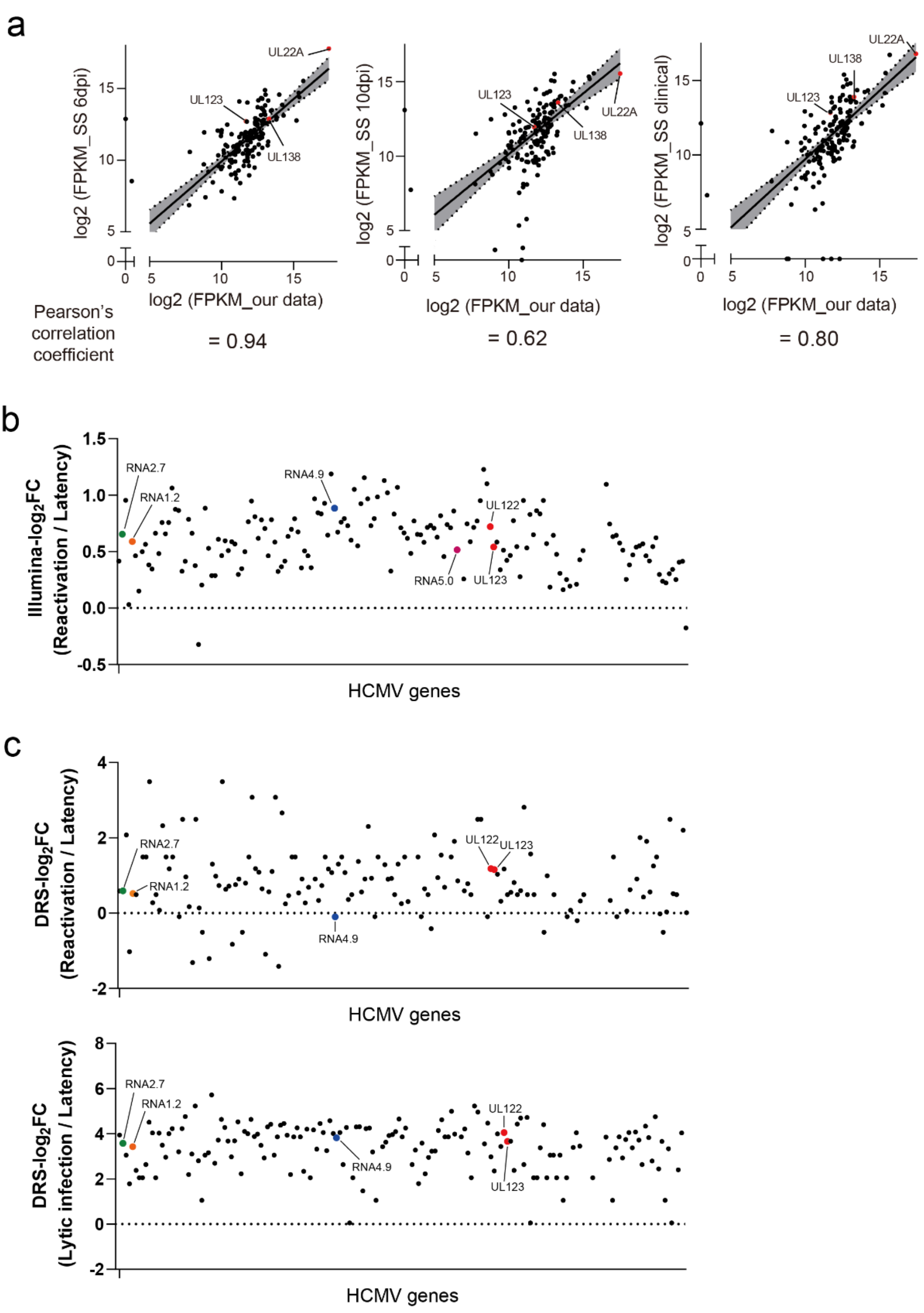

Supplementary Figure 1. Transcriptome analysis of HCMV during latency, reactivation, and lytic infection (a) Comparison of RNA-seq results from the latency sample and the previous dataset (GEO GSE99823). Scatter plot shows FPKM value of HCMV genes. Similarity between each dataset was analyzed by Pearson's correlation coefficient and gray region indicates a 0.95 level of confidence. The sum of viral gene FPKM from each dataset is a million. (b-c) Differential expression of HCMV genes between infectious states. Log2 ratio of fold change in FPKM value from RNA-seq (b) and RPM value from nanopore DRS (c) are shown. HCMV genes without reads in both infectious states are excluded.

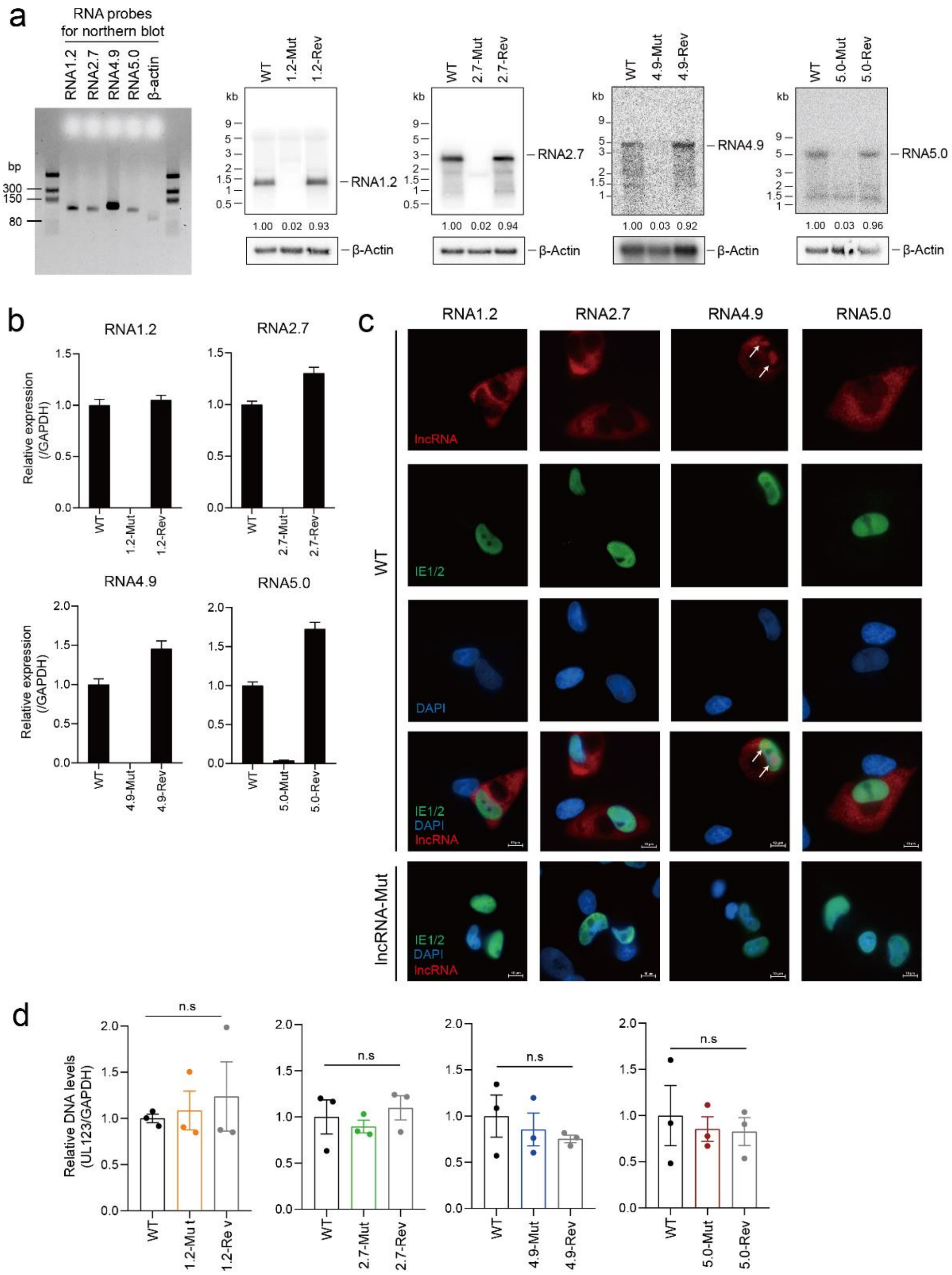

Supplementary Figure 2. LncRNA expression and entry level of recombinant HCMV in lytic infection. (a) Northern blot of HCMV lncRNAs. (Left) HCMV lncRNA targeting RNA probes for northern blot analysis. RNA probes generated by *in vitro* transcription and DNase I digestion were loaded on ethidium bromide stained agarose gel. (Right) Primary HFFs were infected with WT, lncRNA-Mut, or lncRNA-Rev HCMV. Total RNA was extracted and subjected to northern blot analysis using antisense probes for each lncRNA. β-actin served as a loading control. Fold induction was normalized to the WT by quantifying the band intensity of the blots and calculating the ratio of each lncRNA signal to β-actin signal using imageJ software. (b) RNA used in Supplementary Fig. 2a was subjected to qRT-PCR. (c) Subcellular localization of HCMV lncRNAs using WT- and lncRNA-Mut-infected HFFs at 3 d post-infection (3 MOI). LncRNA targeting Cy5-probes (red) were used for smFISH, and FITC-IE1/2 antibodies (green) were used in IFA. Nuclei were stained with DAPI (blue). White arrows in the RNA4.9 smFISH image represent the viral replication complex (VRC). (d) Degree of entry for each virus. At 3 h post-infection, the level of viral DNA in HFFs was measured by qPCR. All experiments were performed in triplicate and data represent mean ± SEM of independent experiments. Statistical significance was calculated by two-tailed unpaired *t*-test.

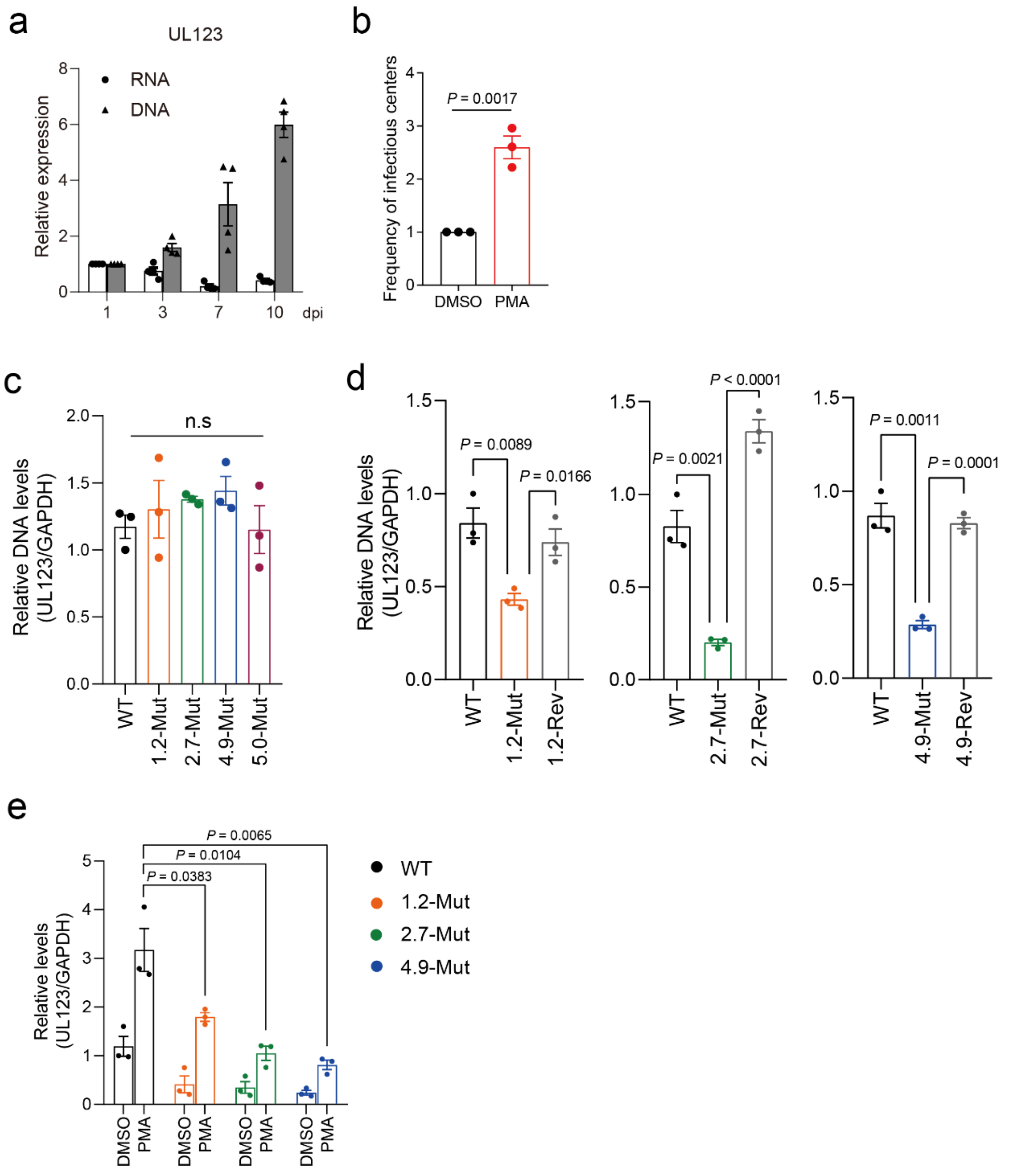

Supplementary Figure 3. Roles of HCMV lncRNAs in latency and reactivation. (a-b) Latency and reactivation of HCMV Toledo in Kasumi-3 cells. Kasumi-3 cells were infected with WT HCMV Toledo at an MOI of 1 and analyzed at the indicated time points. (a) Viral RNA or DNA levels were quantified by detecting UL123 via RT-PCR or qPCR, respectively. (b) Infected Kasumi-3 cells were treated with DMSO or 20 nM of PMA at 10 d post-infection and incubated for 2 d. The cells were then co-cultured with HFFs for 14 d, and relative frequency of infectious centers was calculated by ELDA. (c) Infiltration level of each virus. One hour after spin infection, the level of viral DNA within Kasumi-3 cells was measured by qPCR. (d) Viral DNA level of each virus at 10 d post-infection was measured by qPCR. (e) Infected Kasumi-3 cells were treated with DMSO or 20 nM of PMA at 10 d post-infection and incubated for 2 d. RNA level of UL123 was measured by RT-PCR. Data represent mean ± SEM of 4 (a) or 3 independent experiments (b-e). Statistical significance was calculated by two-tailed unpaired *t*-test.

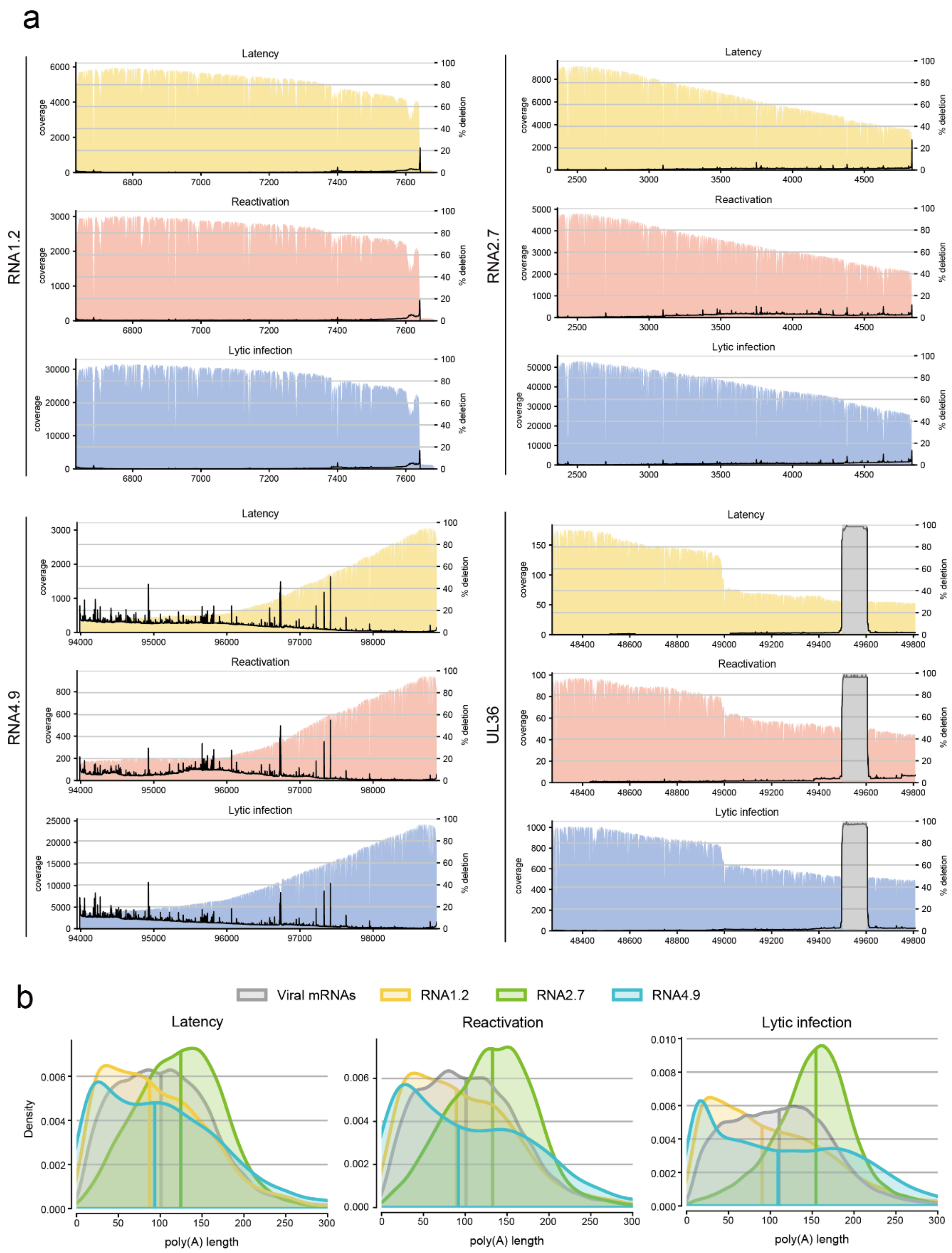

Supplementary Figure 4. Transcript variant form and poly(A) length of HCMV lncRNAs. (a) Total coverage and percentage of splicing by lncRNAs or UL36 at each infection state. Known intronic regions of UL36 are marked with the gray boxes. (b) Poly(A) length distributions of individual lncRNAs and other viral mRNAs in the DRS data from latency, reactivation, and lytic infection samples. Median of each distribution is indicated with a vertical line.

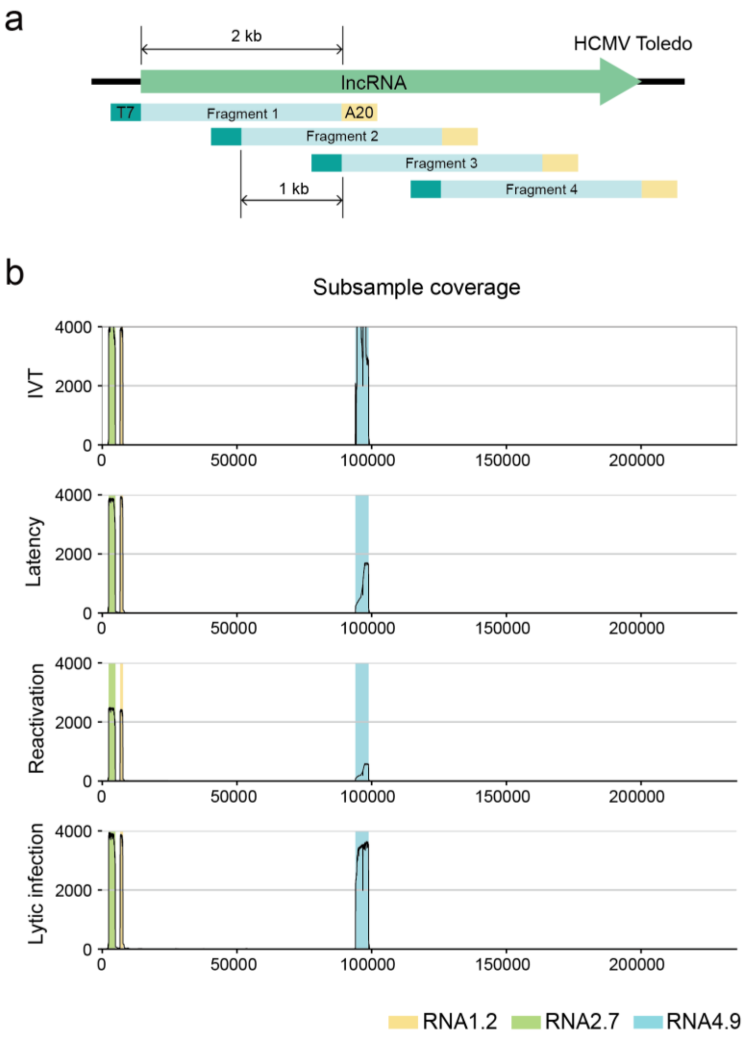

Supplementary Figure 5. *In vitro* transcription of HCMV lncRNAs. (a) Scheme of *in vitro* transcription of HCMV lncRNAs. RNA fragments were about 1–2 kb in length each and exhibited overlapping sequences to cover entire lncRNAs. (b) Subsample coverage of HCMV lncRNAs from DRS reads in latency, reactivation, lytic infection, and IVT. The reads from each library were downsampled so that samples for each infection state or IVT has equal coverage.

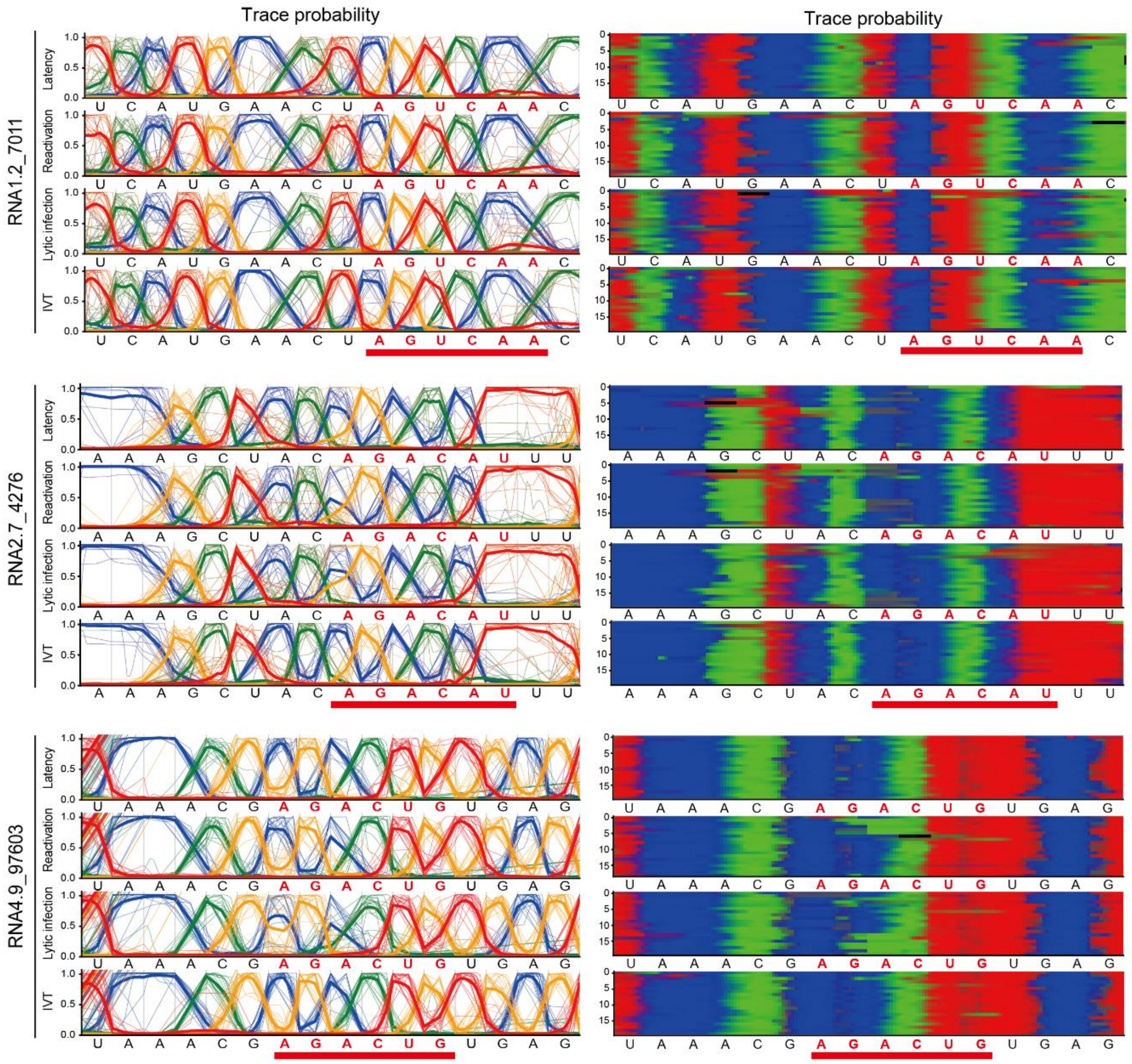

Supplementary Figure 6. Trace probability of each base near genomic positions 7011 (RNA1.2), 4276 (RNA2.7), and 97603 (RNA4.9). Traces from 20 randomly sampled reads were extracted and 1D interpolated with a 10 point / 1 base resolution. The posterior probabilities for A, U, G, and C are plotted with blue, red, yellow, and green lines, respectively (left). Interpolated traces from each base are plotted, and median probabilities are denoted by bold lines (left). The composite RGB (red, green, blue) heatmap describes the posterior probabilities for A, C, and U, represented by blue, green, and red channels, respectively. 'AGACH' motif instances are indicated with underline.

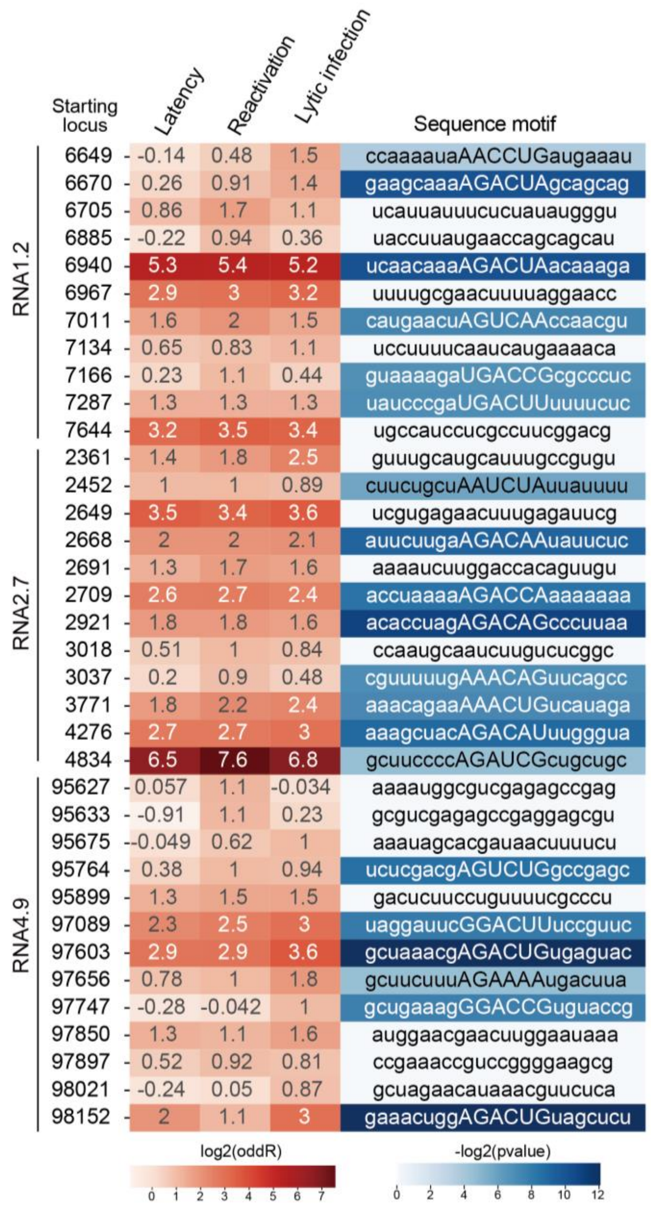

Supplementary Figure 7. Log_2 odds ratio of the putative modified sites of lncRNAs (left) and sequences around the modification sites (right). Sequences mapped to the AGACH motif are presented with p-values of alignment, and aligned motifs are denoted by uppercase letters.

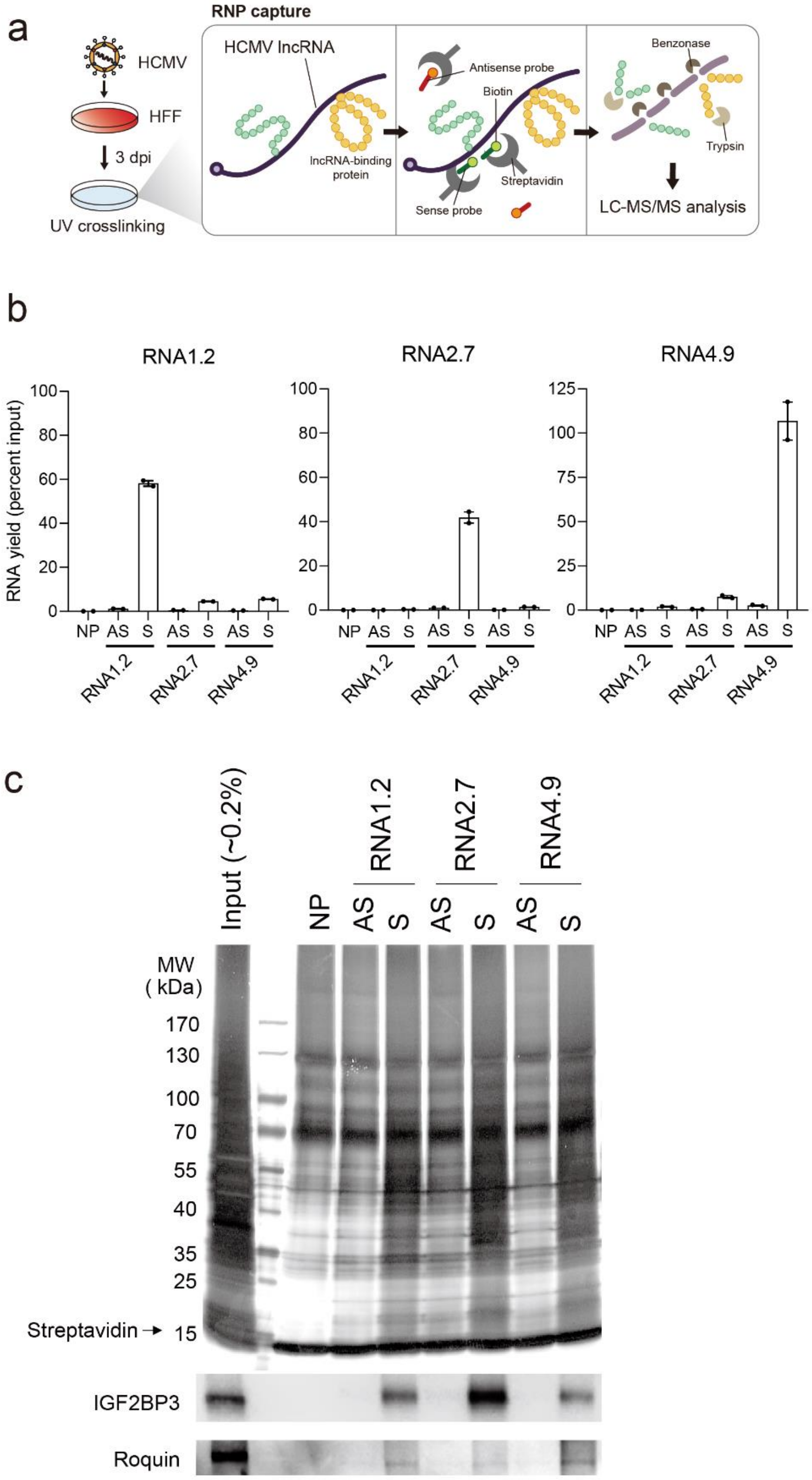

Supplementary Figure 8. RNP complex of HCMV lncRNAs captured by modified RAP-MS. (a) Schematic representation of modified RAP-MS for investigation of HCMV lncRNA-binding proteins. (b) LncRNA yield in modified RAP-MS experiments (n = 2). Data represent mean ± SEM of the percentage of input. (NP, no probe; AS, antisense probe; S, sense probe). (c) Silver staining and immunoblot assay of IGF2BP3 and Roquin (RC3H1) in modified RAP-MS samples. Streptavidin bead background was indicated.

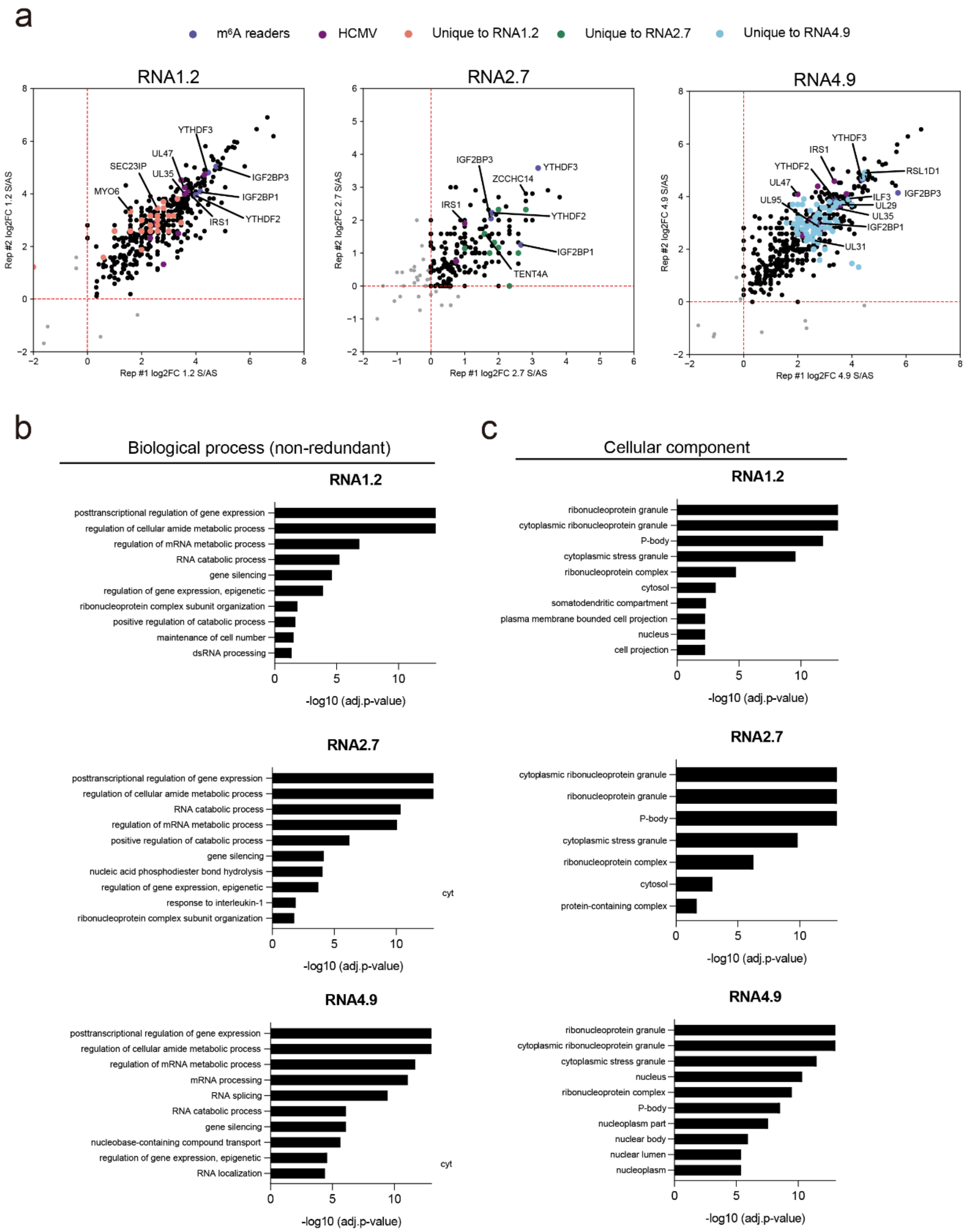

Supplementary Figure 9. Analysis of HCMV lncRNA interactome detected by LC-MS/MS. (a) Enrichment of proteins in independent experiments (n = 2). Scatter plot shows $\log_2$ fold change in MS count of the sense probe (S) versus the antisense probe control (AS). (b-c) GO terms of significantly enriched proteins in each lncRNA were analyzed using a web-based gene set analysis toolkit (Webgestalt.org). GO terms of non-redundant biological processes (b) and cellular components (c) are shown with $-\log_{10}$ (adjusted p-values).

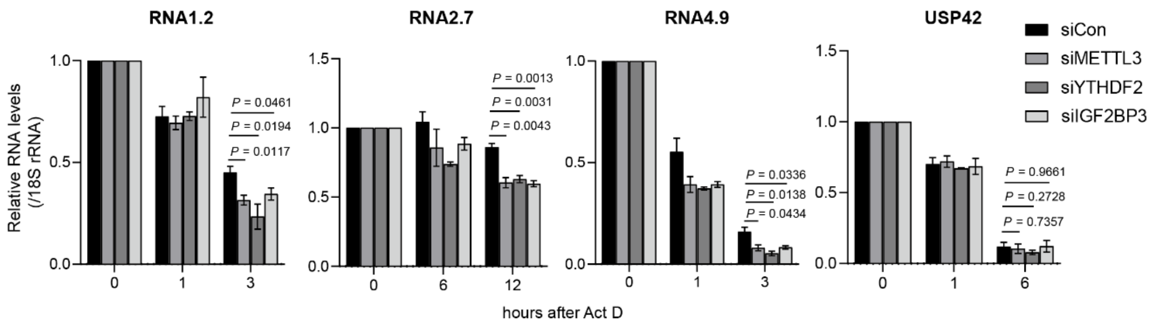

Supplementary Figure 10. RNA1.2, RNA2.7, and RNA4.9 are stabilized by m[6]A machineries. RNA level of each transcript after actinomycin D treatment was measured by qRT-PCR. Data represents values relative to a 0 h sample. USP42 served as a negative control. Data represent mean ± SEM of independent experiments (n = 4), and statistical significance was calculated by two-tailed unpaired *t*-test.

Source data

Figure 2C

Raw western blot images of the viral protein expressions (IE1/2, UL44, and pp28) in WT, lncRNA-Mut, and lncRNA-Rev HCMV infected fibroblasts. The images were obtained by FUSION software.

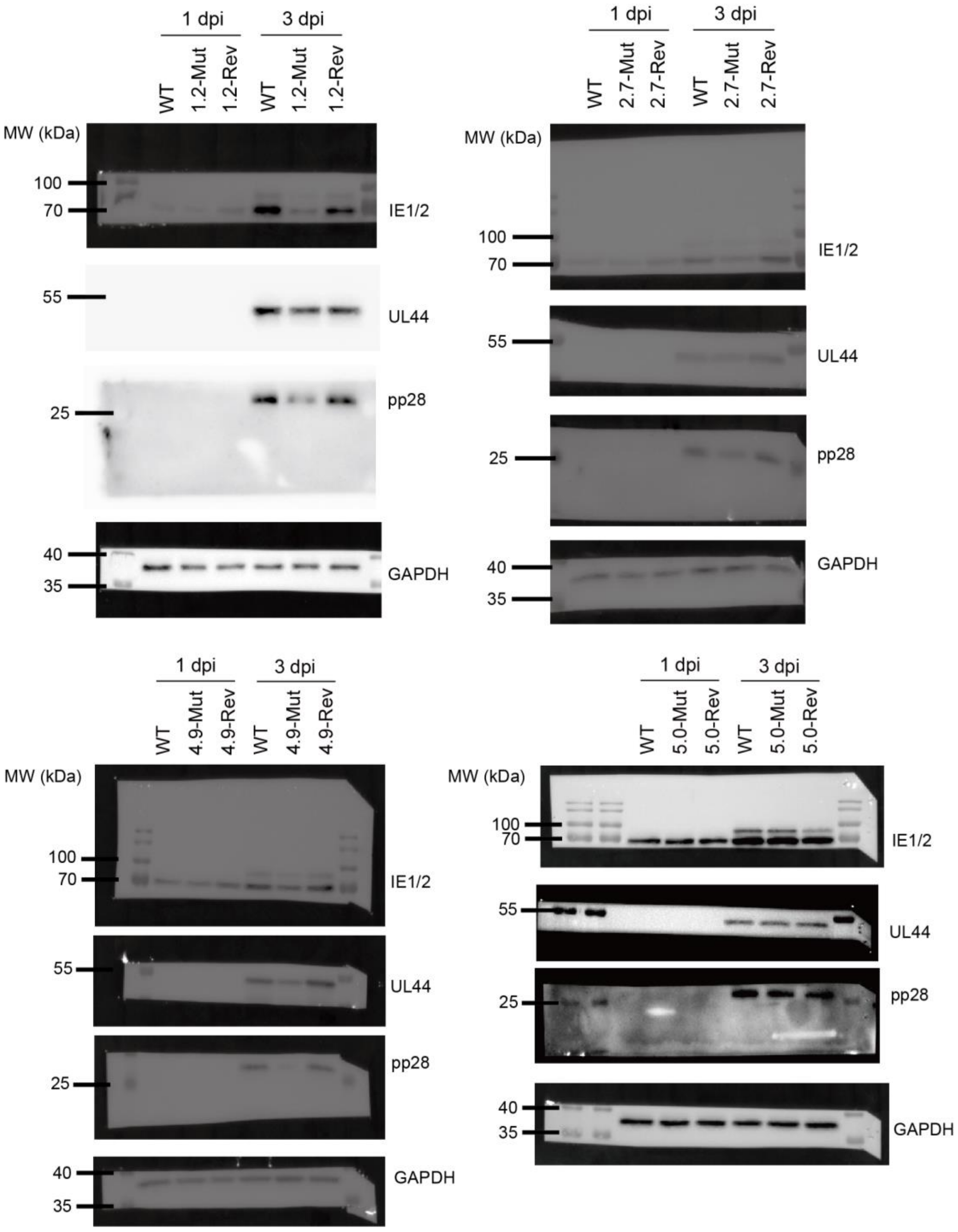

Figure S2A

Raw northern blot images of viral lncRNA expressions in WT, lncRNA-Mut, and lncRNA-Rev HCMV infected fibroblasts. The images were obtained by developing the membrane after hybridization with the probes by Typhoon FLA 7000. Boundaries of the original images were indicated as red solid lines, and the membrane edges were indicated as black dotted lines. Completely overlapped red solid lines and the black dotted lines indicates that the original image boundary and the membrane edge are the same. In this case, the original images were developed by defining membrane containing region only.

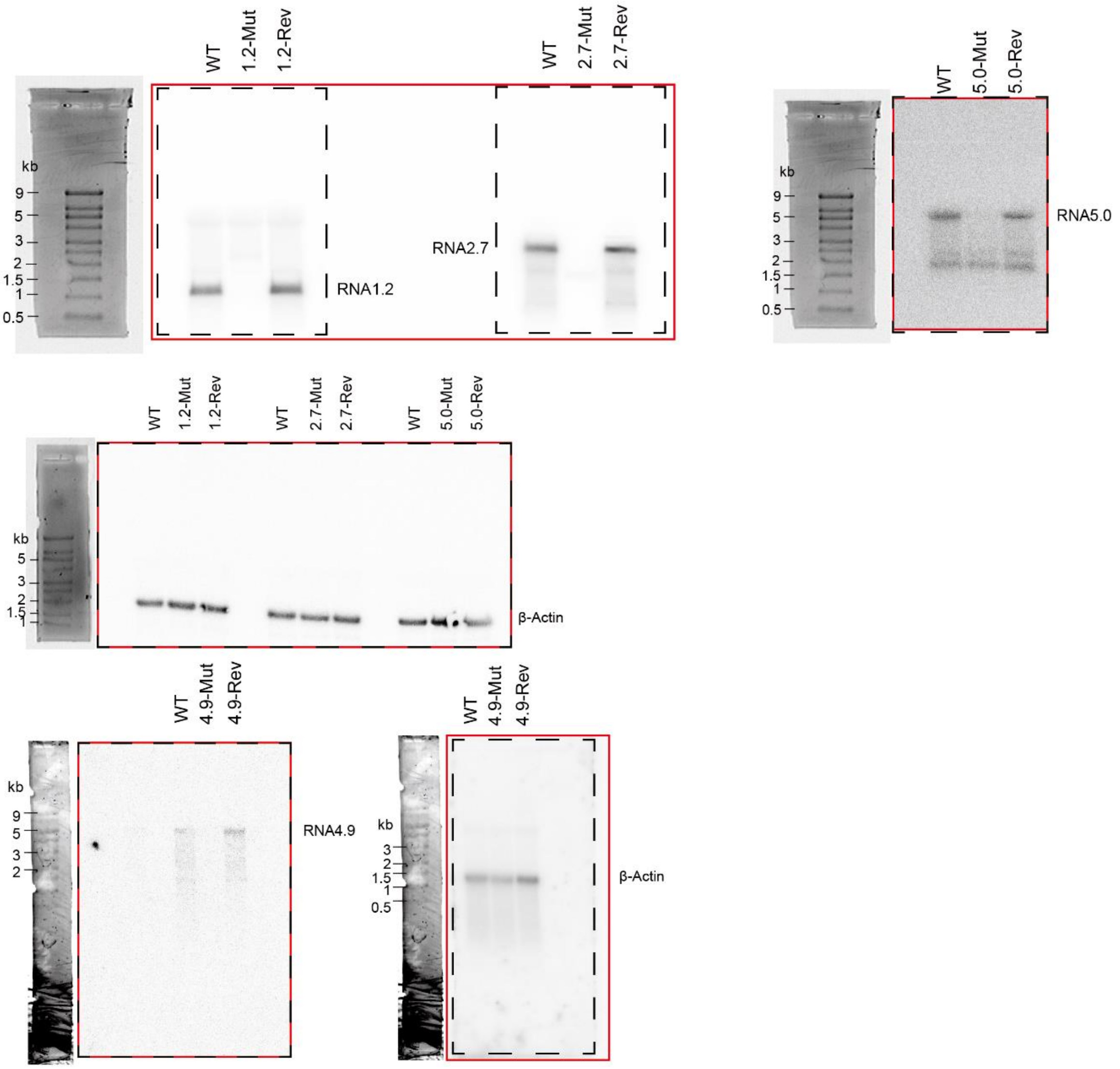

Figure S8C

Uncropped gel image and raw western blot images of viral lncRNA binding proteins. The gel image and the western blot images were obtained by silver staining and by FUSION software, respectively.

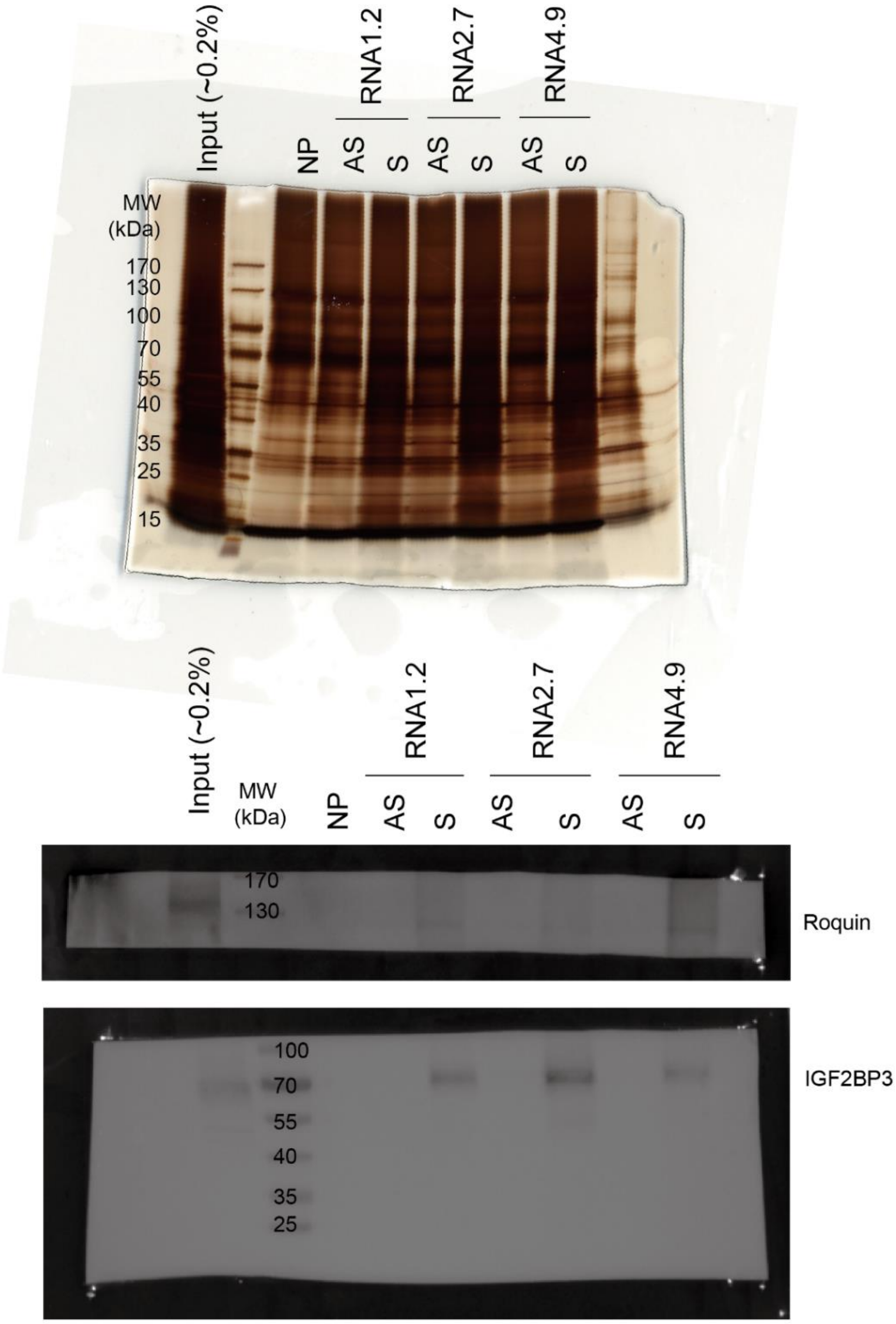

Figure 5D

Raw western blot images of the m^6A machinery protein expressions (METTL3, YTHDF2, and IGF2BP3) in the fibroblasts treated with siRNA targeting each protein. The images were obtained by FUSION software.

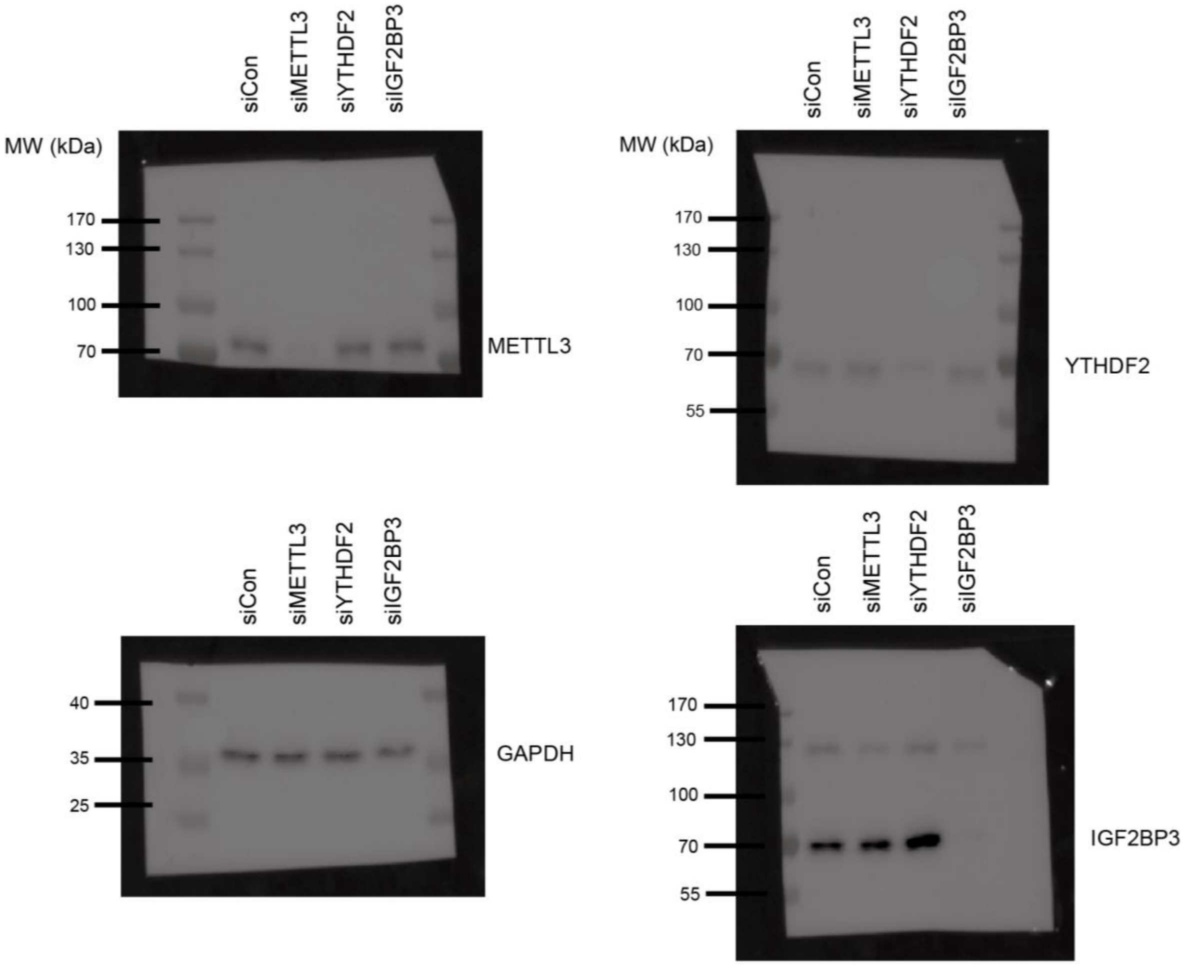

Supplement: Supplementary file 3 — Supplementary Information 3. [file 41598_2022_23317_MOESM3_ESM.pdf]
